# High Hospitalization Rates in Survivors of Childhood Cancer: A Longitudinal Follow-Up Study Using Medical Record Linkage

**DOI:** 10.1371/journal.pone.0159518

**Published:** 2016-07-19

**Authors:** Elske Sieswerda, Anna Font-Gonzalez, Johannes B. Reitsma, Marcel G. W. Dijkgraaf, Richard C. Heinen, Monique W. Jaspers, Helena J. van der Pal, Flora E. van Leeuwen, Huib N. Caron, Ronald B. Geskus, Leontien C. Kremer

**Affiliations:** 1 Department of Pediatric Oncology, Emma Children’s Hospital / Academic Medical Center, Amsterdam, the Netherlands; 2 Julius Center for Health Sciences and Primary Care, University Medical Center, Utrecht, the Netherlands; 3 Clinical Research Unit, Academic Medical Center, Amsterdam, the Netherlands; 4 Center for Human Factors Engineering of Health Information Technology (HITlab), Department of Medical Informatics, Academic Medical Center, Amsterdam, the Netherlands; 5 Department of Medical Oncology, Academic Medical Center, Amsterdam, the Netherlands; 6 Department of Epidemiology, Netherlands Cancer Institute, Amsterdam, the Netherlands; 7 Department of Clinical Epidemiology, Biostatistics and Bioinformatics, Academic Medical Center, Amsterdam, the Netherlands; Taipei Medical University, TAIWAN

## Abstract

Hospitalization rates over time of childhood cancer survivors (CCS) provide insight into the burden of unfavorable health conditions on CCS and health care resources. The objective of our study was to examine trends in hospitalizations of CCS and risk factors in comparison with the general population. We performed a medical record linkage study of a cohort of 1564 ≥five-year CCS with national registers. We obtained a random sample of the general population matched on year of birth, gender and calendar year per CCS retrieved. We quantified and compared hospitalization rates of CCS and reference persons from 1995 until 2005, and we analyzed risk factors for hospitalization within the CCS cohort with multivariable Poisson models. We retrieved hospitalization information from 1382 CCS and 25583 reference persons. The overall relative hospitalization rate (RHR) was 2.2 (95%CI:1.9–2.5) for CCS compared to reference persons. CCS with central nervous system and solid tumors had highest RHRs. Hospitalization rates in CCS were increased compared to reference persons up to at least 30 years after primary diagnosis, with highest rates 5–10 and 20–30 years after primary cancer. RHRs were highest for hospitalizations due to neoplasms (10.7; 95%CI:7.1–16.3) and endocrine/nutritional/metabolic disorders (7.3; 95%CI:4.6–11.7). Female gender (P<0.001), radiotherapy to head and/or neck (P<0.001) or thorax and/or abdomen (P = 0.03) and surgery (P = 0.01) were associated with higher hospitalization rates in CCS. In conclusion, CCS have increased hospitalization rates compared to the general population, up to at least 30 years after primary cancer treatment. These findings imply a high and long-term burden of unfavorable health conditions after childhood cancer on survivors and health care resources.

## Introduction

More than 75% of children with cancer become long-term survivors [1,2]. However, childhood cancer survivors (CCS) are at increased risk of unfavorable health conditions associated with their previous cancer treatment [3–8]. Among CCS aged 45 years, the cumulative prevalence estimates for serious/disabling or life-threatening chronic health conditions is 80% [5,7,9]. It is likely that late effects of cancer treatment in steadily growing numbers of CCS do not only burden the survivors themselves but also the health care system [10].

Hospitalization rates over time provide insight into the burden of unfavorable health conditions on individuals and on health care resources [11–15]. Thus far, previous studies focused on hospital admissions as a measure of burden of disease in CCS but these studies only determined first hospitalization or average hospitalization rates [16–27]. Although these studies provide important insight into the long-term morbidity of CCS, no study has analyzed hospitalization rates in CCS longitudinally by taking into account all hospitalizations within one individual. Such trends are likely to be a better measure for the total burden of unfavorable health conditions [15,28]. Insight into trends and risk factors for all hospitalizations can help to further focus and prioritize long-term follow-up care for CCS at risk of unfavorable health conditions requiring hospitalizations.

Our objective was to determine trends in hospitalization rates of CCS and associated risk factors in comparison with a random reference sample from the general population. To obtain these data, we performed medical record linkage between a study cohort of CCS and Dutch administrative registers.

## Materials and Methods

### Study population and linkage procedure

The Emma Children’s Hospital/Academic Medical Center (EKZ/AMC) childhood cancer survivor cohort is a single-center cohort study of CCS who survived at least five years since primary cancer diagnosis. Details of this study have been described previously [7,29]. Eligibility criteria were a primary childhood cancer diagnosis between 1966 and 1999 before age 18, survival at least five years since diagnosis and being alive at Jan 1^st^ 1995 (N = 1564). From our database, we retrieved information on patient characteristics, cancer diagnosis, all cancer treatment before the date of five-year survival, recurrences and subsequent cancers. Written informed consent was obtained from all childhood cancer patients treated in the EKZ/AMC. The Institutional Review Board of the EKZ/AMC in Amsterdam reviewed and approved the data collection for our cohort register and the study was deemed as evaluation of patient care and was therefore exempt from the need for ethical approval. In addition, after medical record linkage of the cohort to national registers the data did not include directly identifiable variables anymore and therefore data were analyzed anonymously.

Because an individual identification number is lacking in the Hospital Discharge Register (Dutch acronym: LMR), we used a two-step record linkage approach to obtain hospitalization information from CCS. We described and validated the two-step record linkage of our cohort separately [30]. Using this approach we linked 1477 of 1564 eligible CCS to the Municipal Personal Records Database (Dutch acronym: GBA), and subsequently 1382 CCS to LMR.

We additionally obtained a random reference sample of the Dutch general population (20 persons at maximum from GBA with corresponding year of birth and gender per CCS retrieved in GBA), to whom we assigned the starting date of follow-up of the corresponding CCS, i.e. five years after the date of primary cancer diagnosis of the CCS. After record linkage to LMR, the study population consisted of 1382 CCS and of 26583 matched reference persons (both 94% of individuals retrieved from GBA) [30].

### Outcome definition

We assessed the total number of hospitalizations and the total time at risk in CCS and reference persons from 1995 until 2005. We retrieved the primary diagnosis of the hospitalizations coded according to International Classification of Disease version 9 –clinical modification (ICD9-CM) [31]. The LMR contains electronic information on all hospitalizations (day case admissions and clinical hospitalizations) of almost every hospital in the Netherlands from 1995 onwards (coverage >99% until 2004 and 96.7% in 2005) [32]. Uncomplicated (day case) hospitalizations for delivery are not included in the register.

Accrual of time at risk for hospitalization began at the date of five-year survival (i.e. five years after the date of primary childhood cancer diagnosis) or January 1, 1995, whichever came later. Accrual of time at risk ended at January 1, 2006. We only counted time at risk when a person was a unique individual in GBA based on gender, date of birth and postal code[30]. Time during hospitalization was also excluded from the time at risk. When multiple unique periods were available, we summed up the time of the unique periods to define the total time at risk for hospitalization.

We hypothesized that CCS having a primary cancer recurrence or ongoing primary cancer therapy beyond the date of five-year survivorship would have increased exposure to cancer treatment and would simultaneously inflate the hospitalization rate. Because our interest was in hospitalizations beyond primary cancer survival, we censored these CCS and their corresponding reference persons at the date of five-year survival or at the incidence date of first primary cancer recurrence if this happened after the date of five-year survival. Full censoring applied to 90 CCS and partial censoring applied to 35 CCS.

### Statistical analysis

We determined average hospitalization rates during the complete study period of CCS compared to their matched reference persons. We calculated relative hospitalization rates (RHR) and absolute excess risks (AER) of hospitalization per 1000 person-years at risk for the total group. We did the same for CCS categories as well as for specific ICD9-CM hospitalization diagnosis groups. In subsequent analyses, we allowed for changes in hospitalization rates over time in both groups, by including follow-up time since primary cancer diagnosis or attained age as covariate in the regression model. We modeled both covariates via natural cubic splines (using 7 knots) to allow for non-linear trends over time. Parameter estimates of the individual spline components are hard to interpret. Therefore, we used graphs to describe trends. We also estimated hospitalization trends over follow-up time for CCS categories. In all analyses we used a Poisson regression model in which we corrected for recurrent hospitalizations via generalized estimating equations (GEE). We assumed an exchangeable correlation structure.

Finally, we examined risk factors associated with hospitalization rates within the cohort of CCS. In addition to follow-up time since primary cancer diagnosis, we included gender, calendar year of primary cancer diagnosis, age at primary cancer diagnosis and cancer treatments given before the date of five-year survival (cancer surgery; anthracyclines; alkylating agents; other chemotherapy; radiotherapy to the head and/or neck; radiotherapy to the thorax and/or abdomen; radiotherapy to extremities).

We again used a Poisson model, again correcting for recurrent hospitalizations via GEE. We explored effect modification between all included variables and follow-up time, between non-treatment variables (i.e. gender, calendar year of primary cancer diagnosis and age at primary cancer diagnosis) and main cancer treatment modalities (i.e. surgery, chemotherapy, radiotherapy), and between the main cancer treatments themselves. We included them in the final model when statistically significant. We modeled the effects of follow-up time, calendar year of primary cancer diagnosis and age at primary cancer diagnosis via natural cubic splines (using 4 or 5 knots) to allow for a non-linear relationship with the hospitalization rate. Besides providing P-values for the overall effects of the variables, we also present results graphically.

We performed analyses using statistical software R version (2.15.0) using survreg for average RHR and over time, and ggplot2 for the graphs. P values <0.05 were considered statistically significant.

## Results

After medical record linkage, included CCS and reference persons contributed a total of 10,622 and 194,094 years of time at risk respectively (Table 1 and S1 Fig). Median attained ages were 25.7 and 25.9 years. Median duration of follow-up time since (corresponding) date of primary cancer diagnosis was 18.6 years.

**Table 1:** Characteristics of CCS and reference persons contributing to unique follow-up time.

	CCS (n = 1382)		Reference persons (n = 26583)	
	*n*	%	*N*	%
*Gender*				
Male	738	53.4	14347	54.0
Female	644	46.6	12236	46.0
*Year of birth*				
1954–1969	205	14.8	4066	15.3
1970–1985	819	59.3	15462	58.2
1986–1999	358	25.9	7055	26.5
*Calendar year of primary cancer diagnosis*^a^				
1966–1974	117	8.5	2309	8.7
1975–1984	464	33.6	8932	33.6
1985–1994	529	38.3	10037	37.8
1995–1999	272	19.7	5305	20.0
*Age at primary cancer diagnosis*^a^				
Median (range)	6.1	0–17.8	6.0	0–18.4
0–4 yr	607	43.9	11518	43.3
5–9 yr	364	26.3	7197	27.1
10–14 yr	318	23.0	6118	23.0
15–18 yr	93	6.7	1750	6.6
*Primary cancer diagnosis*				
Leukemia/lymphoma	624	45.2		
CNS tumor	98	7.1		
Sarcoma	269	19.5		
Other solid tumors	356	25.8		
Other and unspecified tumors	35	2.5		
*Recurrences of primary cancer*				
None	1161	84.0		
Any recurrence	221	16.0		
*Second tumors*				
None	1310	94.8		
Any second tumor	74	5.4		
*Cancer treatment groups*^b^^,^^c^				
No chemotherapy/radiotherapy (± surgery)	112	8.1		
Chemotherapy (± surgery)	726	52.5		
Radiotherapy (± surgery)	83	6.0		
Chemotherapy and radiotherapy (± surgery)	460	33.3		
*Specific cancer treatments before five-year survival*^c^^,^^d^				
Anthracyclines	586	42.4		
Alkylating agents	700	50.7		
Other chemotherapy	364	26.3		
Radiotherapy to head and/or neck region	374	27.1		
Radiotherapy to thoracic and/or abdominal region	302	21.9		
Radiotherapy to extremities^e^	92	6.7		
*Vital status at the end of follow-up according to GBA*				
Living	1334	96.5	26491	99.7
Deceased	48	3.5	92	0.3
*Attained age at the end of follow-up*				
Median	25.3		25.3	
Range	5.9–51.3		6.1–52.0	
*Follow-up time since (corresponding*^a^*) date of primary cancer diagnosis*				
Median	18.6		18.6	
Range	5.0–39.8		5.7–39.8	
*Years at risk for hospitalization (1995–2005)*				
Sum	10,622		194,094	
Median	8.8		8.1	
Range	0.1–11.0		0.0–11.0	

Abbreviations: CCS: childhood cancer survivors; n: number; GBA: Dutch acronym for Municipal Personal Records Database

^a^ Corresponding date of primary cancer diagnosis of a CCS was assigned to matching reference persons in order to analyze data per survival year (starting at the 5th) and to adjust for calendar period and age.

^b^ Cancer treatment groups were mutually exclusive, i.e. persons could contribute to one cell only. Treatment categories were irrespective of surgical treatment.

^c^ We took all cancer treatment that was given before the date of five-year survival into account.

^d^ Totals add up to more than 1382 because of overlapping categories

^e^ Including 8 CCS with radiotherapy localization defined as “other”.

After applying the censoring for cancer treatment for primary cancer (recurrences) beyond five year survival, we identified 1736 hospitalizations in 1292 CCS, with an average rate of 172 hospitalizations per 1000 person-years. The hospitalization rate in matched reference persons was 79 per 1000 person-years. See S1 Methods for more details on the results of censoring.

Table 2 shows average RHR and AER of CCS compared to reference persons. The overall RHR and AER were 2.2 (95%CI:1.9–2.5) and 93.3 per 1000 person-years at risk, respectively. RHRs and AERs were increased in all CCS cancer diagnosis and treatment categories, with the highest RHRs for CCS originally diagnosed with primary central nervous system (CNS) tumors (RHR:3.4;95%CI:2.7–4.4) and other solid tumors RHR:2.6;95%CI:2.0–3.5). CCS not treated with chemotherapy or radiotherapy had a RHR of 2.5 (95%CI:1.5–4.1). Radiotherapy (with or without surgery, without chemotherapy) was associated with the highest RHR (3.4;95%CI:2.2–5.0) compared to reference persons.

**Table 2:** Average hospitalization rates, relative hospitalization rates and absolute excess rates in CCS and matched reference persons.

	CCS^a^		Matched reference persons^b^				
	*Hospitalizations*	*Hospitalization rate per 1000 py at risk*	*Hospitalizations*	*Hospitalization rate per 1000 py at risk*	*RHR*	*95%CI*	*AER per 1000 py at risk*
All individuals	1736	*172*.*4*	13765	*79*.*1*	2.2	1.9–2.5	93.3
*Gender*							
Male	693	*129*.*5*	4876	*53*.*1*	2.4	2.0–3.0	76.4
Female	1043	*221*.*0*	8889	*108*.*2*	2.0	1.7–2.4	112.8
*Primary cancer diagnosis*							
Leukemia/lymphoma	530	*120*.*0*	5536	*72*.*8*	1.6	1.4–2.0	46.7
CNS	184	*264*.*3*	939	*77*.*2*	3.4	2.7–4.4	187.1
Sarcomas	369	*185*.*9*	3224	*90*.*8*	2.0	1.5–2.8	95.1
Other solid tumors	563	*210*.*1*	3631	*79*.*3*	2.6	2.0–3.5	130.8
Other and unspecified cancers	90	*329*.*8*	435	*98*.*0*	3.4	2.0–5.5	231.8
*Recurrences of primary cancer before five-year survival*							
None	1436	*159*.*8*	11995	*78*.*6*	2.0	1.8–2.4	81.2
Any	300	*277*.*2*	1770	*82*.*9*	3.3	2.5–4.5	194.3
*Cancer treatment*^c^^,^ ^d^							
No chemotherapy or radiotherapy	180	219.5	1255	88.6	2.5	1.5–4.1	130.9
Chemotherapy	614	119.0	6020	67.8	1.8	1.4–2.2	51.2
Radiotherapy	240	344.6	1188	102.0	3.4	2.2–5.0	242.7
Chemotherapy and radiotherapy	701	206.9	5298	89.4	2.3	2.0–2.7	117.6

^a^ Time at risk in CCS was censored at the date of five-year survival in case of ongoing primary cancer recurrence treatment or at the incidence date of first primary cancer recurrence after the date of five-year survival.

^b^ Up to 20 reference persons were sampled per survivor and categorized into cancer diagnosis and treatment groups according to the corresponding CCS

^c^ Cancer treatment groups were mutually exclusive, i.e. persons could contribute to one cell only. Treatment categories were irrespective of surgical treatment.

^d^ We took all cancer treatment that was given before the date of five-year survival into account.

Abbreviations: CCS: childhood cancer survivors; py: person years; RHR: relative hospitalization rate; CI: confidence interval; AER: absolute excess rate.

Table 3 shows that CCS had significantly higher hospitalization rates in comparison to reference persons for 11 of 20 diagnosis groups, especially for neoplasms (RHR:10.7;95%CI:7.1–16.3,AER:24.2), endocrine/nutritional/metabolic disorders (RHR:7.3;95%CI:4.6–11.7,AER:6.3), diseases of the eye (RHR:4.4;95%CI:2.7–7.3,AER:2.9) and diseases of the circulatory system (RHR:3.5;95%CI:2.4–5.1,AER:4.3).

**Table 3:** Average hospitalization rates, relative hospitalization rates and absolute excess rates for ICD9-CM hospitalization diagnosis groups in CCS and reference persons.

	CCS^a^		Reference persons				
	Hospitalizations	Hospitalization rate per 1000 py at risk	Hospitalizations	Hospitalization rate per 1000 py at risk	RHR	95%CI	AER per 1000 py at risk
*ICD group*							
Infectious and parasitic diseases	14	*1*.*4*	101	*0*.*6*	2.4	1.1–5.1	0.8
Neoplasms	269	*26*.*7*	433	*2*.*5*	10.7	7.1–16.3	24.2
Diseases of blood, blood forming organs and disorders involving immune mechanism	17	*1*.*7*	135	*0*.*8*	2.2	0.7–6.5	0.9
Endocrine, nutritional and metabolic diseases	74	*7*.*4*	174	*1*.*0*	7.3	4.6–11.7	6.3
Mental and behavioral disorders	14	*1*.*4*	161	*0*.*9*	1.5	0.8–2.8	0.5
Diseases of the nervous system	39	*3*.*9*	428	*2*.*5*	1.6	0.7–3.3	1.4
Diseases of the eye and adnexa	38	*3*.*8*	149	*0*.*9*	4.4	2.7–7.3	2.9
Diseases of the ear and mastoid process	28	*2*.*8*	332	*1*.*9*	1.5	0.7–2.9	0.9
Diseases of the circulatory system	61	*6*.*1*	300	*1*.*7*	3.5	2.4–5.1	4.3
Diseases of the respiratory system	89	*8*.*8*	1055	*6*.*1*	1.5	0.9–2.3	2.8
Diseases of the digestive system	125	*12*.*4*	1112	*6*.*4*	1.9	1.3–3.0	6.0
Diseases of the skin and subcutaneous tissue	35	*3*.*5*	335	*1*.*9*	1.8	0.9–3.7	1.6
Diseases of the musculoskeletal system and connective tissue	95	*9*.*4*	1680	*9*.*7*	1.0	0.7–1.3	-0.2
Diseases of the genitourinary system	143	*14*.*2*	911	*5*.*2*	2.7	1.7–4.2	9.0
Pregnancy, childbirth and the puerperium	188	*18*.*7*	3231	*18*.*6*	1.0	0.8–1.3	0.1
Conditions originating in the perinatal period	<10^b^	*-*	<10^b^	*-*	-	-	-
Congenital malformations, deformations and chromosomal abnormalities	34	*3*.*4*	222	*1*.*3*	2.6	1.7–4.2	2.1
Symptoms, signs and abnormal clinical findings not elsewhere specified	173	*17*.*2*	789	*4*.*5*	3.8	2.4–5.9	12.6
Injury, poisoning and other consequences of external causes	88	*8*.*7*	1046	*6*.*0*	1.5	1.1–1.9	2.7
Factors influencing health status and contact with health services	212	*21*.*1*	1167	*6*.*7*	3.1	2.5–3.9	14.3

Abbreviations: CCS: childhood cancer survivors; py: person years; RHR: relative hospitalization rate; CI: confidence interval; AER: absolute excess rate

^a^ Time at risk in CCS was censored at the date of five-year survival in case of ongoing primary cancer recurrence treatment or at the incidence date of first primary cancer recurrence after the date of five-year survival

^b^ Less than 10 units (not shown as per Statistics Netherlands patient confidentiality regulations).

**Fig 1:**
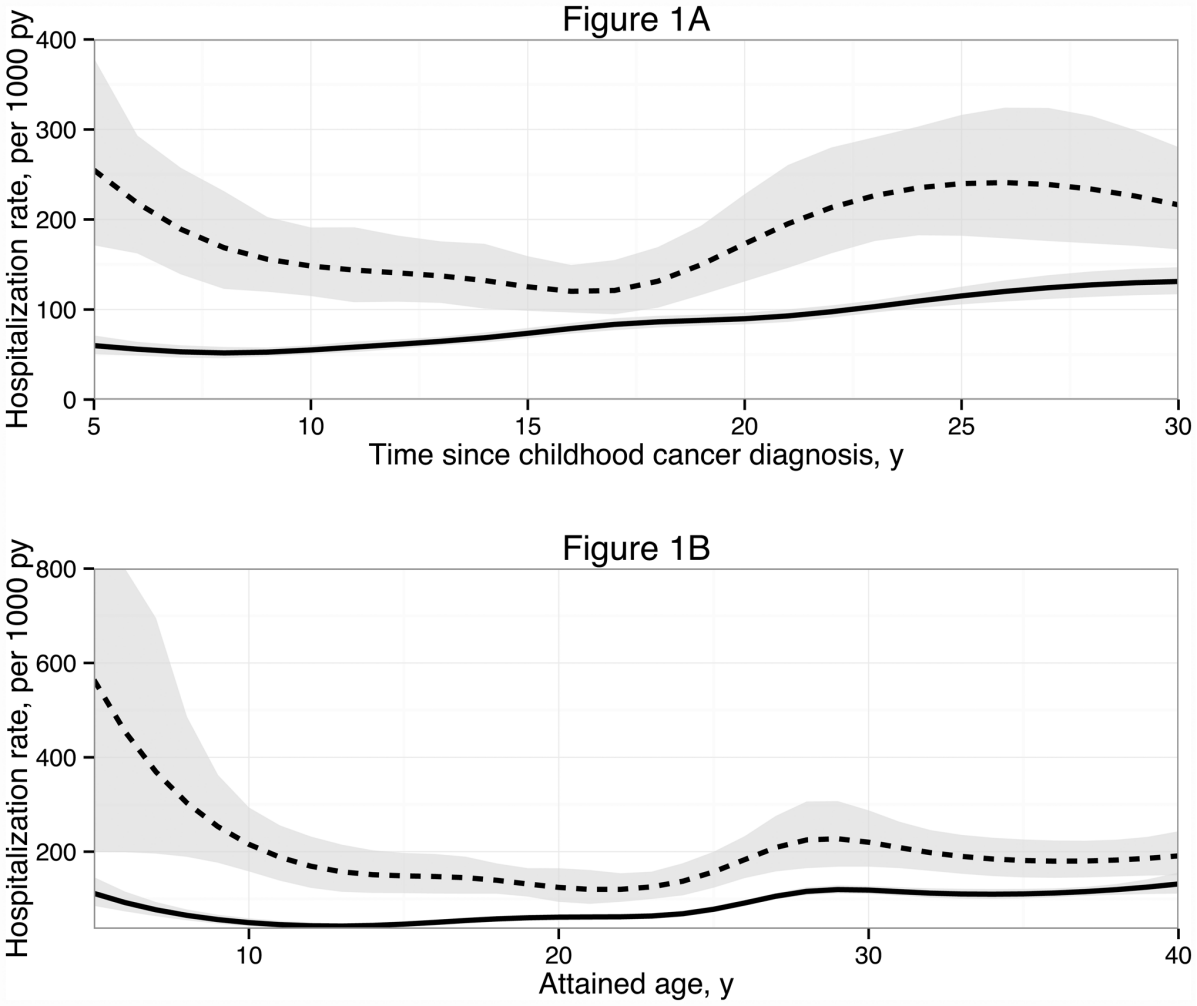
Hospitalization rate of CCS and reference persons over follow-up time since (corresponding) date of primary childhood cancer diagnosis (Fig 1A) and over attained age (Fig 1B). Abbreviations: CCS: childhood cancer survivors; py: person years; y: years. Hospitalization rates per 1000 person years of CCS (dotted line) and reference persons (continuous line) over follow-up time since (corresponding) date of primary childhood cancer diagnosis (Fig 1A) and over attained age (Fig 1B). Grey areas represent 95% confidence intervals. Estimates were made with a Poisson regression model corrected for recurrent hospitalizations. See S1 Table for further information on the differential follow-up time per calendar year of primary cancer diagnosis.

Fig 1 shows the hospitalization rates over follow-up time since (corresponding) date of primary cancer diagnosis (Fig 1a) and attained age (Fig 1b) for CCS and reference persons. Hospitalization rates in CCS were the highest between 5–10 and 20–30 years since primary cancer diagnosis, and before the attained age of 10 and between the attained ages of 25–40 years.

We found generally similar time trends in most other CCS categories, such as in males and females, in groups based on diagnosis, in groups based on recurrence status, and in treatment groups (S2A–S2M Fig).

We identified risk factors for hospitalization within CCS, based on our final multivariate model (Table 4). In this model we included the following significant effect modifiers: follow-up time for gender (P = 0.04), radiotherapy for gender (P = 0.02), follow-up time for surgery (P = 0.06) and calendar year of primary cancer for radiotherapy (P = 0.04). Overall, hospitalization rate was not constant over follow-up time since primary cancer diagnosis (P<0.001). Hospitalization rates were higher in females (P<0.001, non-monotone trend over follow-up time, see S2N Fig), after treatment with surgery (P = 0.01, non-monotone trend over follow-up time, see S2O Fig), after treatment with radiotherapy to thorax and/or abdomen (P = 0.04) and after treatment with radiotherapy to the head and/or neck (P<0.001). Calendar year of primary cancer diagnosis did not significantly influence hospitalization rate (P = 0.08).

**Table 4:** Multivariable model of risk factors for hospitalizations within CCS^a^.

	RHR (95% CI)	P-value	Figure of RHR
*Characteristic*			
Follow-up time since primary cancer diagnosis	-	<0.001	
Gender (female versus male)	Increased risk, non-monotone trend over follow-up time	<0.001	See S2N Fig
Surgery	Increased risk, non-monotone trend over follow-up time (non-significant)	0.01	See S2O Fig
Calendar year of primary cancer diagnosis	-	0.08	
Radiotherapy to thorax and/or abdomen^b^	1.2 (0.9–1.6)	0.04	
Radiotherapy to head and/or neck^b^	1.7 (1.2–2.4)	<0.001	
Radiotherapy to extremities^b^^,^^c^	1.2 (0.8–1.7)	0.08	
Age at primary cancer diagnosis	-	0.17	
Anthracyclines	0.9 (0.7–1.3)	0.72	
Alkylating agents	0.8 (0.6–1.1)	0.12	
Other chemotherapy	0.9 (0.6–1.3)	0.52	

Abbreviations: CCS: childhood cancer survivors; RHR: relative hospitalization rate

^a^ The final model includes the following effect modifiers: follow-up time for gender (P = 0.04), gender for radiotherapy (P = 0.02), follow-up time for surgery (P = 0.06), and calendar year of primary cancer diagnosis for radiotherapy (P = 0.04). P-values in the table correspond to test for overall effect of the respective variables.

^b^ RHR of radiotherapy groups given for the reference calendar year of primary cancer diagnosis (1986) because calendar year is an effect modifier for radiotherapy in the model. No figure provided because the overall effect of calendar year of primary cancer diagnosis was not significant. For the specific RT groups, p-values are based on tests that include the interaction of radiotherapy (any location) with calendar year.

^c^ Including 8 CCS with radiotherapy localization defined as “other”.

## Discussion

We showed that CCS have increased hospitalization rates compared to the general population up to at least 30 years after primary cancer diagnosis. The average hospitalization rate in our CCS cohort was increased 2.2-fold compared to the general population. Especially survivors originally diagnosed with CNS and other solid tumors have an increased hospitalization rate compared to the general population. Within the CCS cohort, we identified that hospitalization rates over follow-up time were higher after treatment with surgery or radiotherapy and in females. These findings imply an increased and long-term burden of unfavorable health conditions after childhood cancer on survivors and health care resources. Since we only studied health conditions that lead to hospitalization, the true burden of unfavorable health conditions after childhood cancer is likely to be even higher.

It is concerning that, after an initial decline in hospitalization rate, we observed a late enhanced increase between the 20^th^ and 30^th^ follow-up year since primary cancer diagnosis compared to the general population. Although we are limited by a hospital register that does not contain electronic data before 1995, likely explanations for this trend could be the deterioration of existing health conditions in CCS as well as new, late onset health conditions.

We found increased hospitalization rates for several hospitalization diagnosis groups including neoplasms, endocrine disorders, circulatory diseases and diseases of the eye in CCS compared to the general population. These numbers imply that several well-known adverse events in CCS translate into an increased risk of hospitalization. The increased risk of hospitalization for neoplasms will be mainly due to secondary neoplasms since we applied censoring at late primary cancer recurrences and treatment. The increased risk of secondary neoplasms in CCS is well known and primarily related to radiotherapy [33,34]. The high risk of endocrine disorders and circulatory diseases in CCS has also been described and is mainly related to local radiotherapy as well as several chemotherapeutic agents [21–23,35–42]. The risk of diseases of the eye is known, but less often described in CCS. Likely explanations for this increased hospitalization rate are diseases such as cataract after radiotherapy and other problems after orbital tumors, retinoblastoma and glucocorticoids, in combination with a low background risk of (hospitalization for) eye disease in the general population [43,44]. Finally, the increased rates in the diagnosis groups “factors influencing health status and contact with health services” and “symptoms, signs and abnormal findings not elsewhere specified” could be explained by clinical signs and symptoms in CCS unusual for the age range and low-threshold clinical evaluations because of anxiety for cancer recurrence. Future studies should explore the underlying diagnoses of the hospitalization diagnosis groups.

The main treatment-related risk factors for hospitalization within CCS were surgery and radiotherapy, especially head and/or neck irradiation. These findings confirm previous study findings that irradiated survivors are the most vulnerable CCS risk group [45]. CCS treated with surgery only have generally been treated with extensive surgery. We confirmed previous findings within our cohort that this treatment modality is associated with an increased burden of health conditions [7]. In our analyses within the CCS cohort we found that female survivors are at increased risk of hospitalization compared to male survivors. Female gender has been previously linked to unfavorable health conditions in CCS. However, we found that males had a higher relative risk but a lower excess risk of hospitalization than females when comparing hospitalizations of CCS to the general population for both sexes [46]. Thus, the increased risk in hospitalization among females CCS in our analyses might be partly due to increased risks of hospitalization in females in the general population in this age range.

Other studies that used hospital discharge registers and determined hospitalization rates in CCS in general found increased risks of hospitalization [16,19,20]. Reported risks were an odds ratio (OR) for any hospitalization of 4.4 [16], a standardized hospitalization ratio (SHR) of any hospitalization of 2.8 [19] and a hospital admission rate ratio of 1.7 per year [20]. These studies were not able to determine hospitalization rates in CCS longitudinally by taking into account all hospitalizations within one individual and did not include multivariate detailed treatment-related risk factor analyses. Two other studies have used questionnaires to determine average hospitalization rates in CCS [17,18]. These studies compared hospitalizations rates to available reference rates obtained from existing surveys. Both studies also found an increased risk of hospitalization in CCS (OR 1.6 and relative rate 1.9 respectively). They found an increased risk of hospitalization for CNS tumors [18] female survivors and after radiotherapy [17].

The most important strength of our study is that we determined hospitalization rates over follow-up time by taking into account repeated hospitalizations within one individual. Using this approach, we showed that hospitalization rates differ over time and show a clinically relevant late enhanced increase between the 20^th^ and 30^th^ follow-up year since primary cancer diagnosis. We were also able to link hospitalizations to complete information on an individual’s previous cancer treatment. Our analysis accounted for recurrent hospitalizations, as well as for the confounding influences of age, gender, calendar period and late recurrences of primary cancer. Without applying the censoring at late primary cancer recurrences in our analyses, the average RHR would have increased substantially to 2.8, primarily due to hospitalizations in the first 5 to 10 years since cancer diagnosis (data not shown). Finally, hospitalizations in our study were prospectively registered in a national administrative register in the same way for CCS and reference persons. We therefore had an appropriate reference group, no risk of selection bias due to (non-)response and low risk of differential misclassification of the outcome.

A limitation of our study is that we could not directly link CCS to the hospital discharge register with one unique person identifier. However, by using unique time at risk based on linkage parameters, we analyzed hospitalization rates over follow-up time in a valid manner [30]. Another limitation is the differential follow-up for survivors treated in different time periods in our study. The data of CCS diagnosed in the 1990’s will have contributed mostly to the 5–15 years follow-up time, while the data of CCS diagnosed in the 1970’s contributed mostly to the ≥20 years follow-up time (S1 Table). However, when we added calendar year of primary cancer diagnosis to the model, this effect was not significant and the effect of follow-up time did not change much (S2P Fig). Because of the differential follow-up within our study design, we could not further explore whether the effect of follow-up time differed by calendar year of diagnosis. Nevertheless, we accounted for our longitudinal design with left-truncation by including the entry-time in our statistical analyses [47]. Finally, for this study we rely on correct hospitalization registration by Dutch hospitals, although this applies to both CCS and reference persons and has been found to be acceptable by others [48] and by ourselves [30].

## Conclusions

We showed that CCS have increased hospitalization rates compared to the general population up to many years after reaching adulthood, especially survivors originally diagnosed with CNS and other solid tumors. CCS treated with surgery or radiotherapy are at highest risk for hospitalization. Further refinements in the trends of hospitalization over time and evaluation of disease specific hospitalization rates in relation to cancer treatments are needed. The high and long-term burden of unfavorable health conditions on CCS and on health care resources underscores the need for awareness and knowledge about these health conditions among survivors and health care professionals.

## Supporting Information

S1 Fig Flowchart of individuals included in the EKZ/AMC cohort of CCS and the sampled reference population from the GBA. ^1^ We censored CCS (and corresponding reference persons) who developed a first primary cancer recurrence after the date of five-year survival (n = 86) and with ongoing cancer therapy for a primary cancer recurrence at the date of five-year survival (n = 39). Complete censoring applied to 90 CCS (51 of the 86 CCS who developed a primary cancer recurrence before 1995 plus the 39 CCS). Abbreviations: EKZ/AMC: Emma Children’s Hospital/Academic Medical Center; CCS: childhood cancer survivors; *N*: number; GBA: Municipal Personal Records Database (In Dutch: Gemeentelijke Basisadministratie).
(TIF)

S2 Fig **(A) Hospitalization rate of male CCS and reference persons over follow-up time.** Hospitalization rates per 1000 person years of CCS (dotted line) and reference persons (continuous line) over follow-up time since (corresponding) date of primary childhood cancer diagnosis. Grey areas represent 95% confidence intervals. Estimates were made with a Poisson regression model corrected for recurrent hospitalizations. Abbreviations: CCS: childhood cancer survivors; py: person years; y: years. Please note that numbers of hospitalizations differed between categories and that we adjusted the y-axes accordingly in the figures. **(B) Hospitalization rate of female CCS and reference persons over follow-up time.** Hospitalization rates per 1000 person years of CCS (dotted line) and reference persons (continuous line) over follow-up time since (corresponding) date of primary childhood cancer diagnosis. Grey areas represent 95% confidence intervals. Estimates were made with a Poisson regression model corrected for recurrent hospitalizations. Abbreviations: CCS: childhood cancer survivors; py: person years; y: years. Please note that numbers of hospitalizations differed between categories and that we adjusted the y-axes accordingly in the figures. **(C) Hospitalization rate of CCS previously diagnosed with leukemia or lymphoma and reference persons over follow-up time.** Hospitalization rates per 1000 person years of CCS (dotted line) and reference persons (continuous line) over follow-up time since (corresponding) date of primary childhood cancer diagnosis. Grey areas represent 95% confidence intervals. Estimates were made with a Poisson regression model corrected for recurrent hospitalizations. Abbreviations: CCS: childhood cancer survivors; py: person years; y: years. Please note that numbers of hospitalizations differed between categories and that we adjusted the y-axes accordingly in the figures. **(D) Hospitalization rate of CCS previously diagnosed with a central nervous system tumor and reference persons over follow-up time.** Hospitalization rates per 1000 person years of CCS (dotted line) and reference persons (continuous line) over follow-up time since (corresponding) date of primary childhood cancer diagnosis. Grey areas represent 95% confidence intervals. Estimates were made with a Poisson regression model corrected for recurrent hospitalizations. Abbreviations: CCS: childhood cancer survivors; py: person years; y: years. Please note that numbers of hospitalizations differed between categories and that we adjusted the y-axes accordingly in the figures. **(E) Hospitalization rate of CCS previously diagnosed with a sarcoma and matched reference persons over follow-up time.** Hospitalization rates per 1000 person years of CCS (dotted line) and reference persons (continuous line) over follow-up time since (corresponding) date of primary childhood cancer diagnosis. Grey areas represent 95% confidence intervals. Estimates were made with a Poisson regression model corrected for recurrent hospitalizations. Abbreviations: CCS: childhood cancer survivors; py: person years; y: years. Please note that numbers of hospitalizations differed between categories and that we adjusted the y-axes accordingly in the figures. **(F) Hospitalization rate of CCS previously diagnosed with other solid tumors and reference persons over follow-up time.** Hospitalization rates per 1000 person years of CCS (dotted line) and reference persons (continuous line) over follow-up time since (corresponding) date of primary childhood cancer diagnosis. Grey areas represent 95% confidence intervals. Estimates were made with a Poisson regression model corrected for recurrent hospitalizations. Abbreviations: CCS: childhood cancer survivors; py: person years; y: years. Please note that numbers of hospitalizations differed between categories and that we adjusted the y-axes accordingly in the figures. **(G) Hospitalization rate of CCS previously diagnosed with other and unspecified cancers and reference persons over follow-up time.** Hospitalization rates per 1000 person years of CCS (dotted line) and reference persons (continuous line) over follow-up time since (corresponding) date of primary childhood cancer diagnosis. Grey areas represent 95% confidence intervals. Estimates were made with a Poisson regression model corrected for recurrent hospitalizations. Abbreviations: CCS: childhood cancer survivors; py: person years; y: years. Please note that numbers of hospitalizations differed between categories and that we adjusted the y-axes accordingly in the figures. **(H) Hospitalization rate of CCS without a recurrence before five-year survival and reference persons over follow-up time.** Hospitalization rates per 1000 person years of CCS (dotted line) and reference persons (continuous line) over follow-up time since (corresponding) date of primary childhood cancer diagnosis. Grey areas represent 95% confidence intervals. Estimates were made with a Poisson regression model corrected for recurrent hospitalizations. Abbreviations: CCS: childhood cancer survivors; py: person years; y: years. Please note that numbers of hospitalizations differed between categories and that we adjusted the y-axes accordingly in the figures. **(I) Hospitalization rate of CCS with a recurrence before five-year survival and reference persons over follow-up time.** Hospitalization rates per 1000 person years of CCS (dotted line) and reference persons (continuous line) over follow-up time since (corresponding) date of primary childhood cancer diagnosis. Grey areas represent 95% confidence intervals. Estimates were made with a Poisson regression model corrected for recurrent hospitalizations. Abbreviations: CCS: childhood cancer survivors; py: person years; y: years. Please note that numbers of hospitalizations differed between categories and that we adjusted the y-axes accordingly in the figures. **(J) Hospitalization rate of CCS treated without chemotherapy and radiotherapy (with or without surgery) and reference persons over follow-up time.** Hospitalization rates per 1000 person years of CCS (dotted line) and reference persons (continuous line) over follow-up time since (corresponding) date of primary childhood cancer diagnosis. Grey areas represent 95% confidence intervals. Estimates were made with a Poisson regression model corrected for recurrent hospitalizations. Abbreviations: CCS: childhood cancer survivors; py: person years; y: years. Please note that numbers of hospitalizations differed between categories and that we adjusted the y-axes accordingly in the figures. **(K) Hospitalization rate of CCS treated with chemotherapy and without radiotherapy (with or without surgery) and reference persons over follow-up time.** Hospitalization rates per 1000 person years of CCS (dotted line) and reference persons (continuous line) over follow-up time since (corresponding) date of primary childhood cancer diagnosis. Grey areas represent 95% confidence intervals. Estimates were made with a Poisson regression model corrected for recurrent hospitalizations. Abbreviations: CCS: childhood cancer survivors; py: person years; y: years. Please note that numbers of hospitalizations differed between categories and that we adjusted the y-axes accordingly in the figures. **(L) Hospitalization rate of CCS treated with radiotherapy and without chemotherapy (with or without surgery) and reference persons over follow-up time.** Hospitalization rates per 1000 person years of CCS (dotted line) and reference persons (continuous line) over follow-up time since (corresponding) date of primary childhood cancer diagnosis. Grey areas represent 95% confidence intervals. Estimates were made with a Poisson regression model corrected for recurrent hospitalizations. Abbreviations: CCS: childhood cancer survivors; py: person years; y: years. Please note that numbers of hospitalizations differed between categories and that we adjusted the y-axes accordingly in the figures. **(M) Hospitalization rate of CCS treated with chemotherapy and radiotherapy (with or without surgery) and reference persons over follow-up time.** Hospitalization rates per 1000 person years of CCS (dotted line) and reference persons (continuous line) over follow-up time since (corresponding) date of primary childhood cancer diagnosis. Grey areas represent 95% confidence intervals. Estimates were made with a Poisson regression model corrected for recurrent hospitalizations. Abbreviations: CCS: childhood cancer survivors; py: person years; y: years. Please note that numbers of hospitalizations differed between categories and that we adjusted the y-axes accordingly in the figures. **(N) RHR of female CCS versus male CCS treated with (Yes) or without (No) radiotherapy over follow-up time.** Estimates were made with a Poisson regression model corrected for recurrent hospitalizations. The final model includes the following effect modifiers: follow-up time for gender, radiotherapy for gender, follow-up time for surgery, and calendar year of primary cancer diagnosis for radiotherapy. Other variables included are radiotherapy to thorax and/or abdomen; radiotherapy to head and/or neck; radiotherapy to extremities (including 8 CCS with radiotherapy localization defined as “other”); anthracyclines; alkylating agents; other chemotherapy. Grey areas represent 95% confidence intervals. Abbreviations: RHR: relative hospitalization rate; CCS: childhood cancer survivors; py: person years; y: years. **(O) RHR of CCS treated with versus without surgery over follow-up time.** Estimates were made with a Poisson regression model corrected for recurrent hospitalizations. The final model includes the following effect modifiers: follow-up time for gender, radiotherapy for gender, follow-up time for surgery, and calendar year of primary cancer diagnosis for radiotherapy. Other variables included are radiotherapy to thorax and/or abdomen; radiotherapy to head and/or neck; radiotherapy to extremities (including 8 CCS with radiotherapy localization defined as “other”); anthracyclines; alkylating agents; other chemotherapy. Grey areas represent 95% confidence intervals. After 30 years of follow-up numbers were too small to give appropriate estimates. Abbreviations: RHR: relative hospitalization rate; CCS: childhood cancer survivors; py: person years; y: years. **(P) Hospitalization rate of CCS and reference persons over follow-up time and adjusting for calendar year of primary cancer diagnosis.** Hospitalization rates per 1000 person years of CCS (dotted line) and reference persons (continuous line) over follow-up time since (corresponding) date of primary childhood cancer diagnosis and adjusting for calendar year (the plot presents four calendar year periods: 1995, 1985, 1980 and 1975 from left to right). Grey areas represent 95% confidence intervals. Estimates were made with a Poisson regression model corrected for recurrent hospitalizations. Abbreviations: py: person years; y: years.
(ZIP)

S1 Methods Details on censoring of CCS and corresponding reference persons. (DOCX)

S1 Table Calendar year of primary cancer diagnosis and corresponding follow-up time since primary cancer diagnosis and attained age at the end of follow-up. (DOCX)
