# Supplementary figures and images for: High Hospitalization Rates in Survivors of Childhood Cancer: A Longitudinal Follow-Up Study Using Medical Record Linkage
Source: PLoS One. 2016 Jul 19;11(7):e0159518. doi: 10.1371/journal.pone.0159518 (PMC4951023; doi:10.1371/journal.pone.0159518)

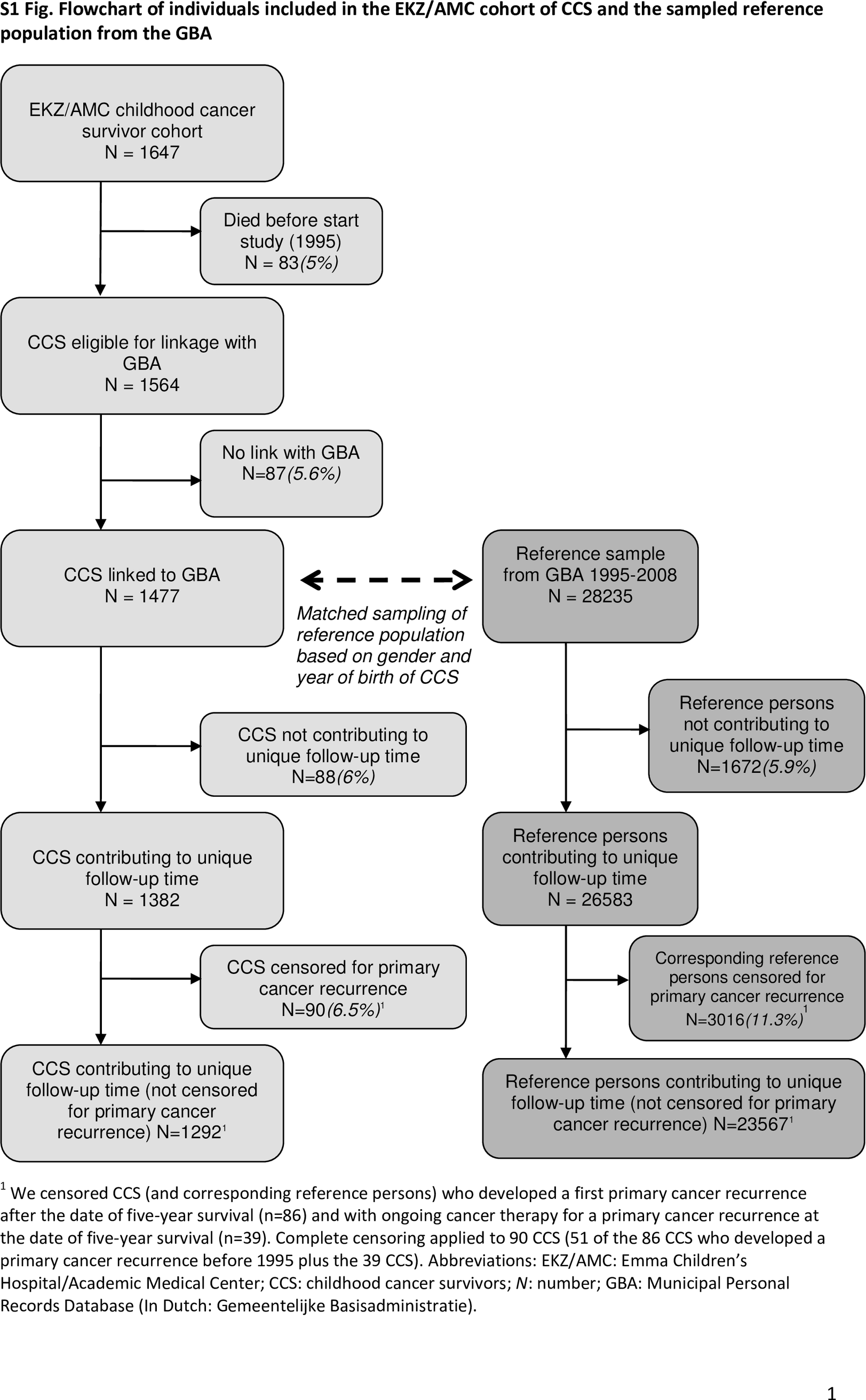

Supplement: S1 Fig — 1 We censored CCS (and corresponding reference persons) who developed a first primary cancer recurrence after the date of five-year survival (n = 86) and with ongoing cancer therapy for a primary cancer recurrence at the date of five-year survival (n = 39). Complete censoring applied to 90 CCS (51 of the 86 CCS who developed a primary cancer recurrence before 1995 plus the 39 CCS). Abbreviations: EKZ/AMC: Emma Children’s Hospital/Academic Medical Center; CCS: childhood cancer survivors; N: number; GBA: Municipal Personal Records Database (In Dutch: Gemeentelijke Basisadministratie). (TIF) [file pone.0159518.s001.tif]

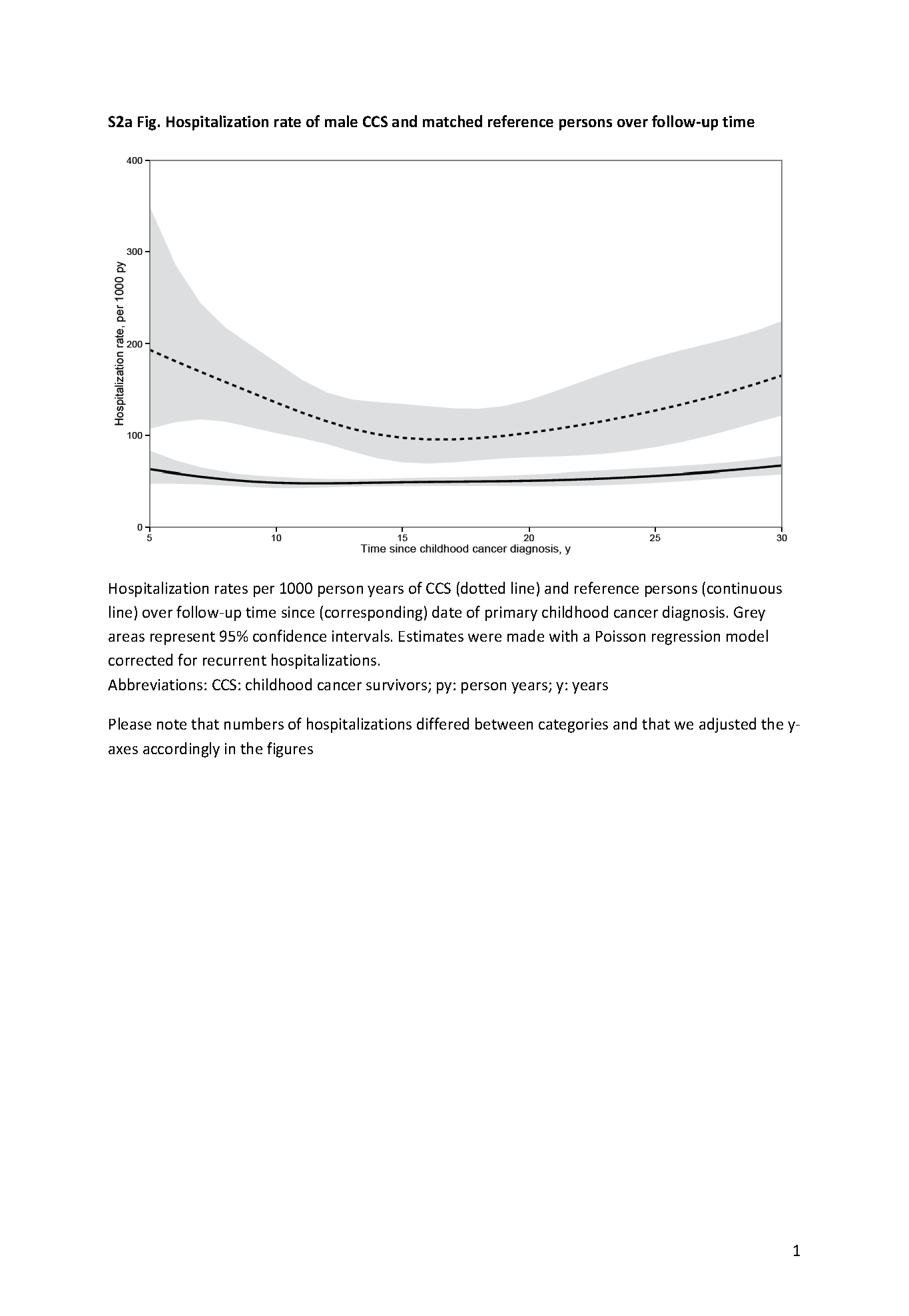

Supplement: S2 Fig — (A) Hospitalization rate of male CCS and reference persons over follow-up time. Hospitalization rates per 1000 person years of CCS (dotted line) and reference persons (continuous line) over follow-up time since (corresponding) date of primary childhood cancer diagnosis. Grey areas represent 95% confidence intervals. Estimates were made with a Poisson regression model corrected for recurrent hospitalizations. Abbreviations: CCS: childhood cancer survivors; py: person years; y: years. Please note that numbers of hospitalizations differed between categories and that we adjusted the y-axes accordingly in the figures. (B) Hospitalization rate of female CCS and reference persons over follow-up time. Hospitalization rates per 1000 person years of CCS (dotted line) and reference persons (continuous line) over follow-up time since (corresponding) date of primary childhood cancer diagnosis. Grey areas represent 95% confidence intervals. Estimates were made with a Poisson regression model corrected for recurrent hospitalizations. Abbreviations: CCS: childhood cancer survivors; py: person years; y: years. Please note that numbers of hospitalizations differed between categories and that we adjusted the y-axes accordingly in the figures. (C) Hospitalization rate of CCS previously diagnosed with leukemia or lymphoma and reference persons over follow-up time. Hospitalization rates per 1000 person years of CCS (dotted line) and reference persons (continuous line) over follow-up time since (corresponding) date of primary childhood cancer diagnosis. Grey areas represent 95% confidence intervals. Estimates were made with a Poisson regression model corrected for recurrent hospitalizations. Abbreviations: CCS: childhood cancer survivors; py: person years; y: years. Please note that numbers of hospitalizations differed between categories and that we adjusted the y-axes accordingly in the figures. (D) Hospitalization rate of CCS previously diagnosed with a central nervous system tumor and [file pone.0159518.s002.zip › S2_Fig/S2a_Fig.tif]

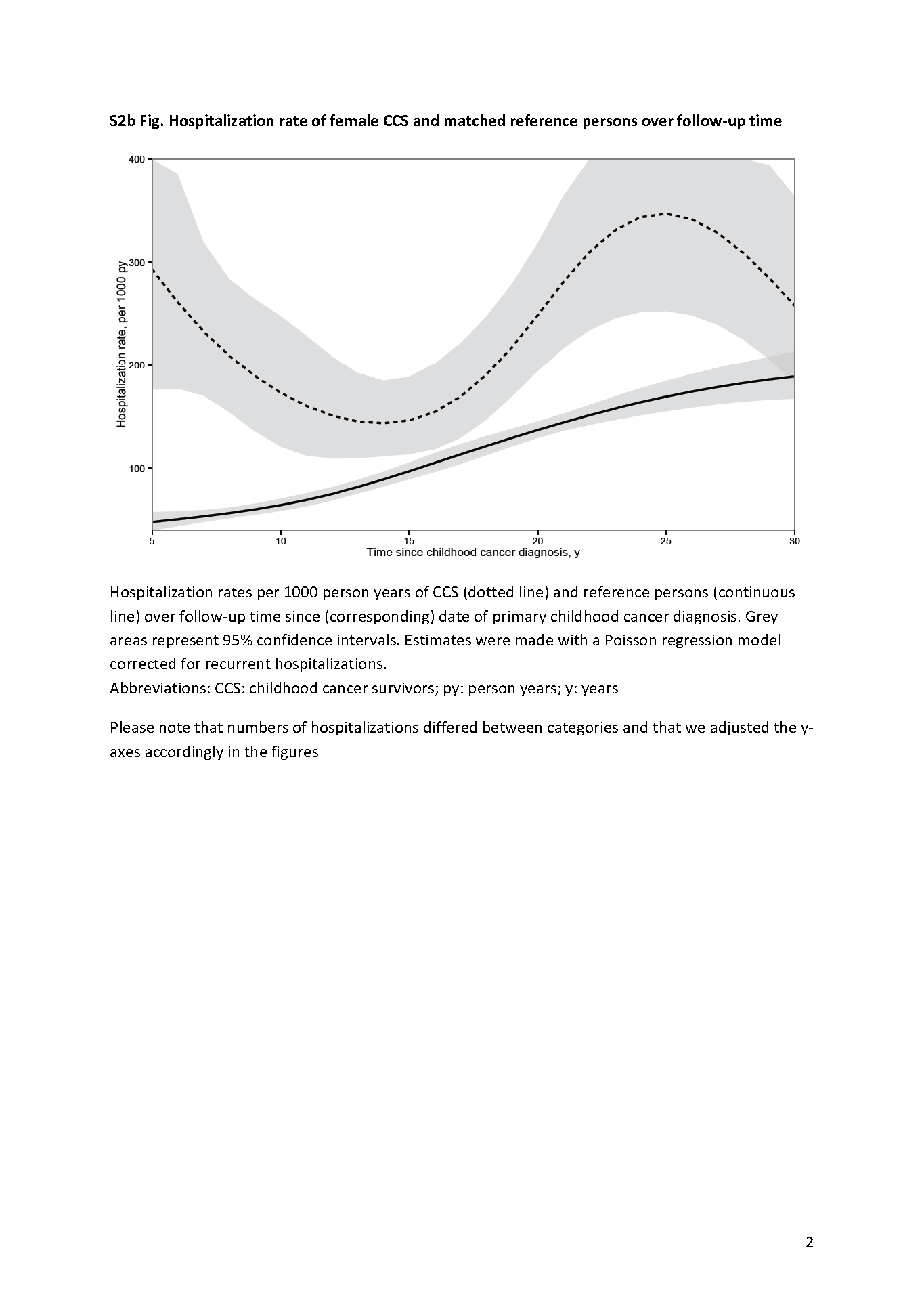

Supplement: S2 Fig — (A) Hospitalization rate of male CCS and reference persons over follow-up time. Hospitalization rates per 1000 person years of CCS (dotted line) and reference persons (continuous line) over follow-up time since (corresponding) date of primary childhood cancer diagnosis. Grey areas represent 95% confidence intervals. Estimates were made with a Poisson regression model corrected for recurrent hospitalizations. Abbreviations: CCS: childhood cancer survivors; py: person years; y: years. Please note that numbers of hospitalizations differed between categories and that we adjusted the y-axes accordingly in the figures. (B) Hospitalization rate of female CCS and reference persons over follow-up time. Hospitalization rates per 1000 person years of CCS (dotted line) and reference persons (continuous line) over follow-up time since (corresponding) date of primary childhood cancer diagnosis. Grey areas represent 95% confidence intervals. Estimates were made with a Poisson regression model corrected for recurrent hospitalizations. Abbreviations: CCS: childhood cancer survivors; py: person years; y: years. Please note that numbers of hospitalizations differed between categories and that we adjusted the y-axes accordingly in the figures. (C) Hospitalization rate of CCS previously diagnosed with leukemia or lymphoma and reference persons over follow-up time. Hospitalization rates per 1000 person years of CCS (dotted line) and reference persons (continuous line) over follow-up time since (corresponding) date of primary childhood cancer diagnosis. Grey areas represent 95% confidence intervals. Estimates were made with a Poisson regression model corrected for recurrent hospitalizations. Abbreviations: CCS: childhood cancer survivors; py: person years; y: years. Please note that numbers of hospitalizations differed between categories and that we adjusted the y-axes accordingly in the figures. (D) Hospitalization rate of CCS previously diagnosed with a central nervous system tumor and [file pone.0159518.s002.zip › S2_Fig/S2b_Fig.tif]

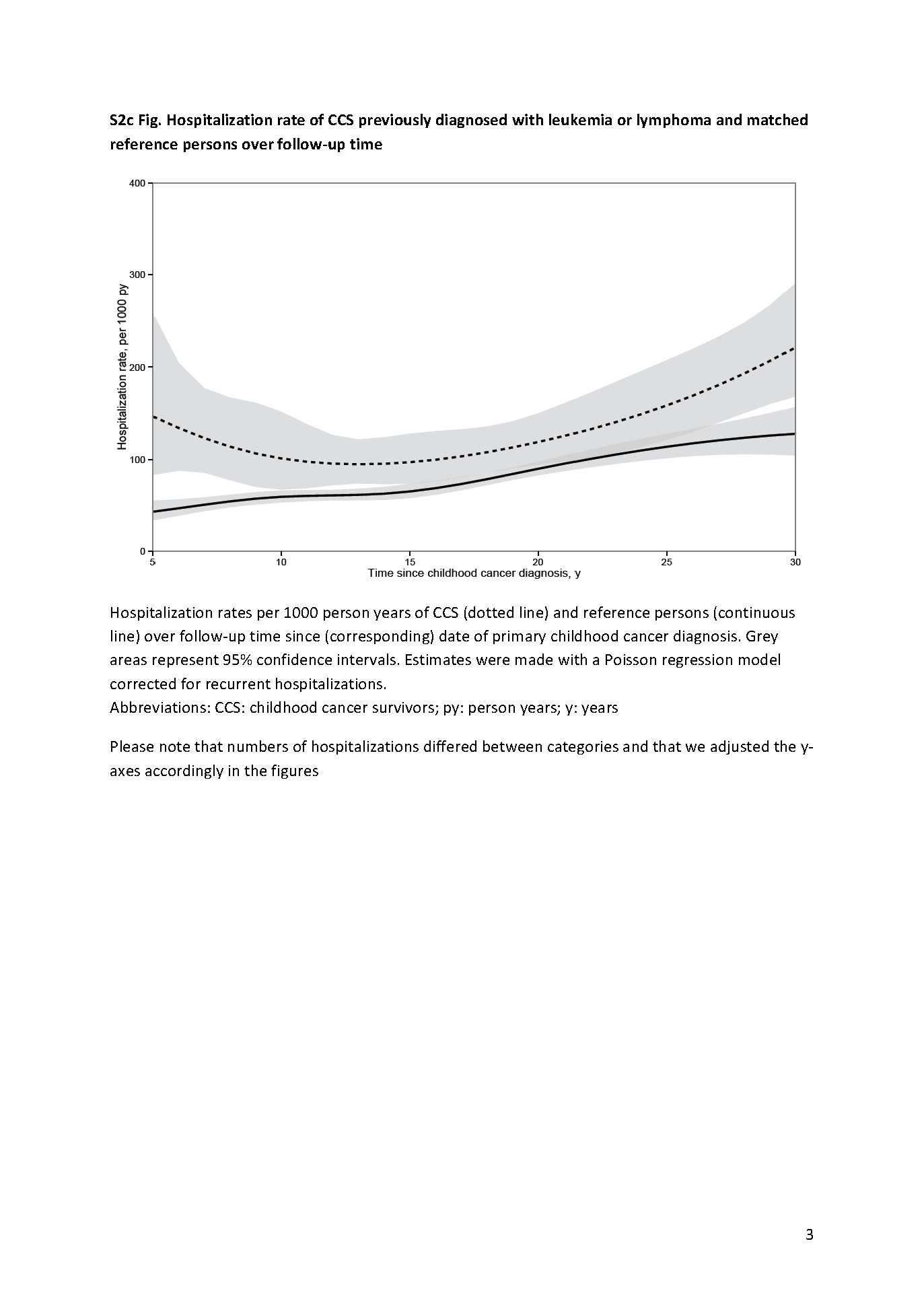

Supplement: S2 Fig — (A) Hospitalization rate of male CCS and reference persons over follow-up time. Hospitalization rates per 1000 person years of CCS (dotted line) and reference persons (continuous line) over follow-up time since (corresponding) date of primary childhood cancer diagnosis. Grey areas represent 95% confidence intervals. Estimates were made with a Poisson regression model corrected for recurrent hospitalizations. Abbreviations: CCS: childhood cancer survivors; py: person years; y: years. Please note that numbers of hospitalizations differed between categories and that we adjusted the y-axes accordingly in the figures. (B) Hospitalization rate of female CCS and reference persons over follow-up time. Hospitalization rates per 1000 person years of CCS (dotted line) and reference persons (continuous line) over follow-up time since (corresponding) date of primary childhood cancer diagnosis. Grey areas represent 95% confidence intervals. Estimates were made with a Poisson regression model corrected for recurrent hospitalizations. Abbreviations: CCS: childhood cancer survivors; py: person years; y: years. Please note that numbers of hospitalizations differed between categories and that we adjusted the y-axes accordingly in the figures. (C) Hospitalization rate of CCS previously diagnosed with leukemia or lymphoma and reference persons over follow-up time. Hospitalization rates per 1000 person years of CCS (dotted line) and reference persons (continuous line) over follow-up time since (corresponding) date of primary childhood cancer diagnosis. Grey areas represent 95% confidence intervals. Estimates were made with a Poisson regression model corrected for recurrent hospitalizations. Abbreviations: CCS: childhood cancer survivors; py: person years; y: years. Please note that numbers of hospitalizations differed between categories and that we adjusted the y-axes accordingly in the figures. (D) Hospitalization rate of CCS previously diagnosed with a central nervous system tumor and [file pone.0159518.s002.zip › S2_Fig/S2c_Fig.tif]

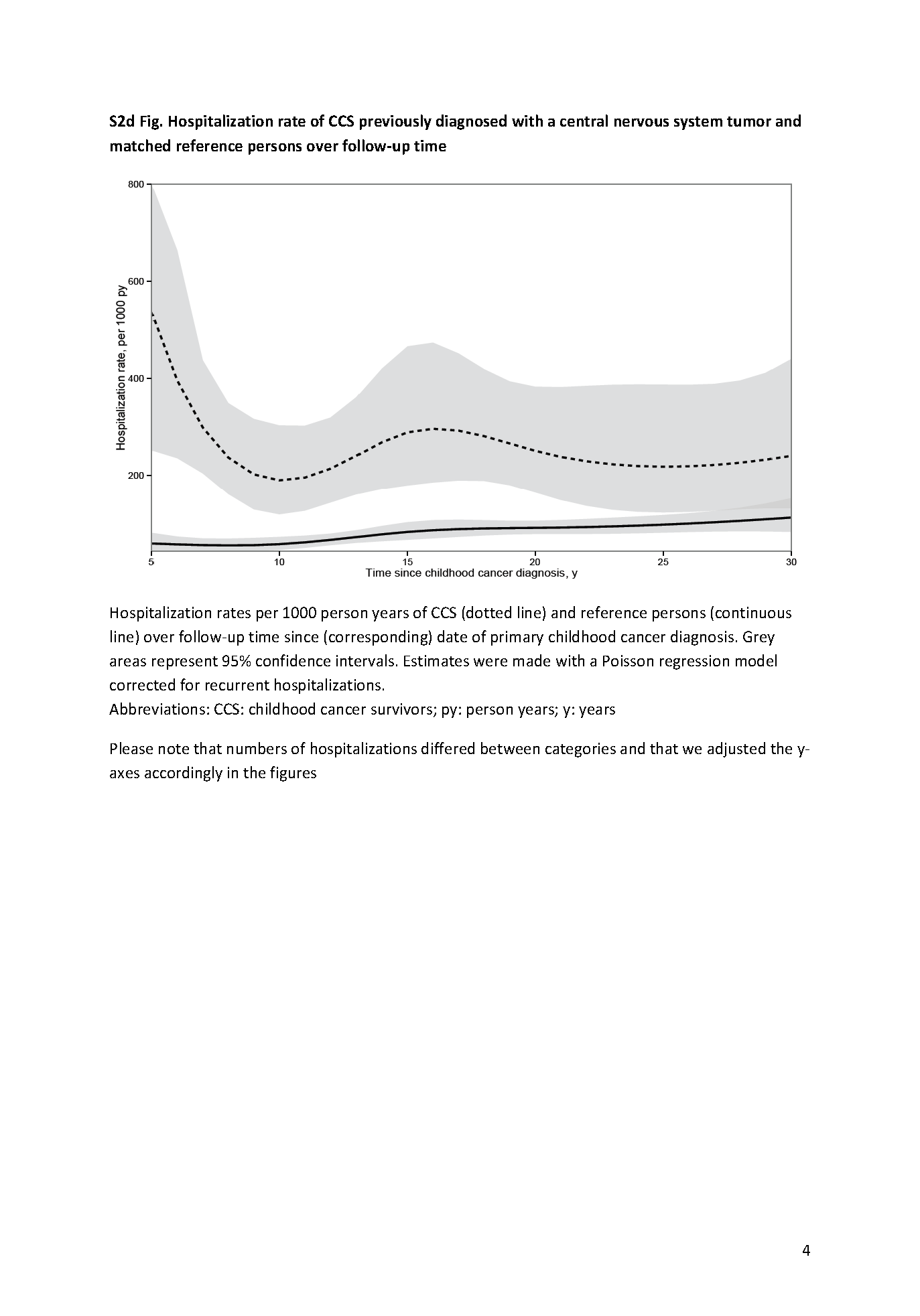

Supplement: S2 Fig — (A) Hospitalization rate of male CCS and reference persons over follow-up time. Hospitalization rates per 1000 person years of CCS (dotted line) and reference persons (continuous line) over follow-up time since (corresponding) date of primary childhood cancer diagnosis. Grey areas represent 95% confidence intervals. Estimates were made with a Poisson regression model corrected for recurrent hospitalizations. Abbreviations: CCS: childhood cancer survivors; py: person years; y: years. Please note that numbers of hospitalizations differed between categories and that we adjusted the y-axes accordingly in the figures. (B) Hospitalization rate of female CCS and reference persons over follow-up time. Hospitalization rates per 1000 person years of CCS (dotted line) and reference persons (continuous line) over follow-up time since (corresponding) date of primary childhood cancer diagnosis. Grey areas represent 95% confidence intervals. Estimates were made with a Poisson regression model corrected for recurrent hospitalizations. Abbreviations: CCS: childhood cancer survivors; py: person years; y: years. Please note that numbers of hospitalizations differed between categories and that we adjusted the y-axes accordingly in the figures. (C) Hospitalization rate of CCS previously diagnosed with leukemia or lymphoma and reference persons over follow-up time. Hospitalization rates per 1000 person years of CCS (dotted line) and reference persons (continuous line) over follow-up time since (corresponding) date of primary childhood cancer diagnosis. Grey areas represent 95% confidence intervals. Estimates were made with a Poisson regression model corrected for recurrent hospitalizations. Abbreviations: CCS: childhood cancer survivors; py: person years; y: years. Please note that numbers of hospitalizations differed between categories and that we adjusted the y-axes accordingly in the figures. (D) Hospitalization rate of CCS previously diagnosed with a central nervous system tumor and [file pone.0159518.s002.zip › S2_Fig/S2d_Fig.tif]

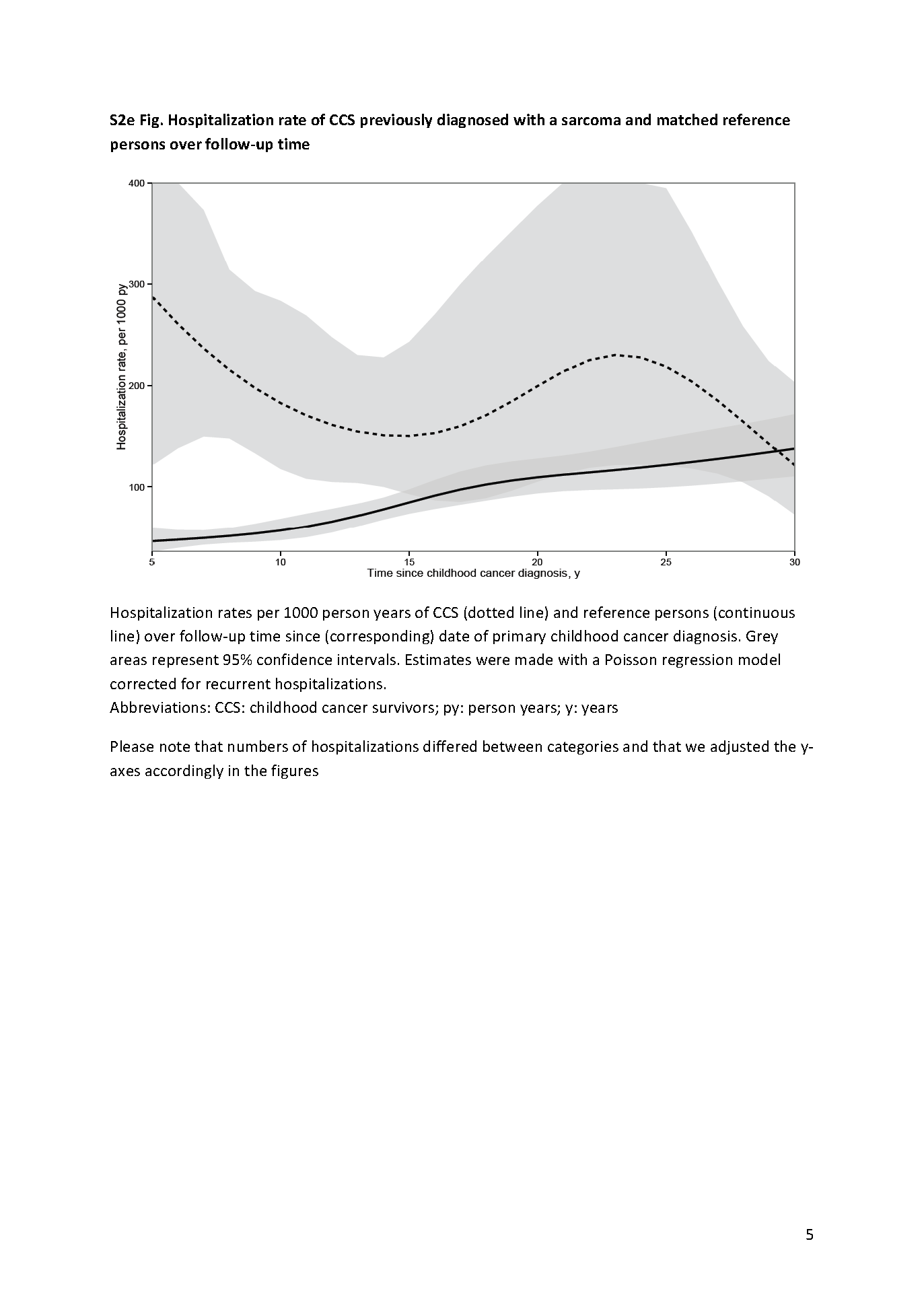

Supplement: S2 Fig — (A) Hospitalization rate of male CCS and reference persons over follow-up time. Hospitalization rates per 1000 person years of CCS (dotted line) and reference persons (continuous line) over follow-up time since (corresponding) date of primary childhood cancer diagnosis. Grey areas represent 95% confidence intervals. Estimates were made with a Poisson regression model corrected for recurrent hospitalizations. Abbreviations: CCS: childhood cancer survivors; py: person years; y: years. Please note that numbers of hospitalizations differed between categories and that we adjusted the y-axes accordingly in the figures. (B) Hospitalization rate of female CCS and reference persons over follow-up time. Hospitalization rates per 1000 person years of CCS (dotted line) and reference persons (continuous line) over follow-up time since (corresponding) date of primary childhood cancer diagnosis. Grey areas represent 95% confidence intervals. Estimates were made with a Poisson regression model corrected for recurrent hospitalizations. Abbreviations: CCS: childhood cancer survivors; py: person years; y: years. Please note that numbers of hospitalizations differed between categories and that we adjusted the y-axes accordingly in the figures. (C) Hospitalization rate of CCS previously diagnosed with leukemia or lymphoma and reference persons over follow-up time. Hospitalization rates per 1000 person years of CCS (dotted line) and reference persons (continuous line) over follow-up time since (corresponding) date of primary childhood cancer diagnosis. Grey areas represent 95% confidence intervals. Estimates were made with a Poisson regression model corrected for recurrent hospitalizations. Abbreviations: CCS: childhood cancer survivors; py: person years; y: years. Please note that numbers of hospitalizations differed between categories and that we adjusted the y-axes accordingly in the figures. (D) Hospitalization rate of CCS previously diagnosed with a central nervous system tumor and [file pone.0159518.s002.zip › S2_Fig/S2e_Fig.tif]

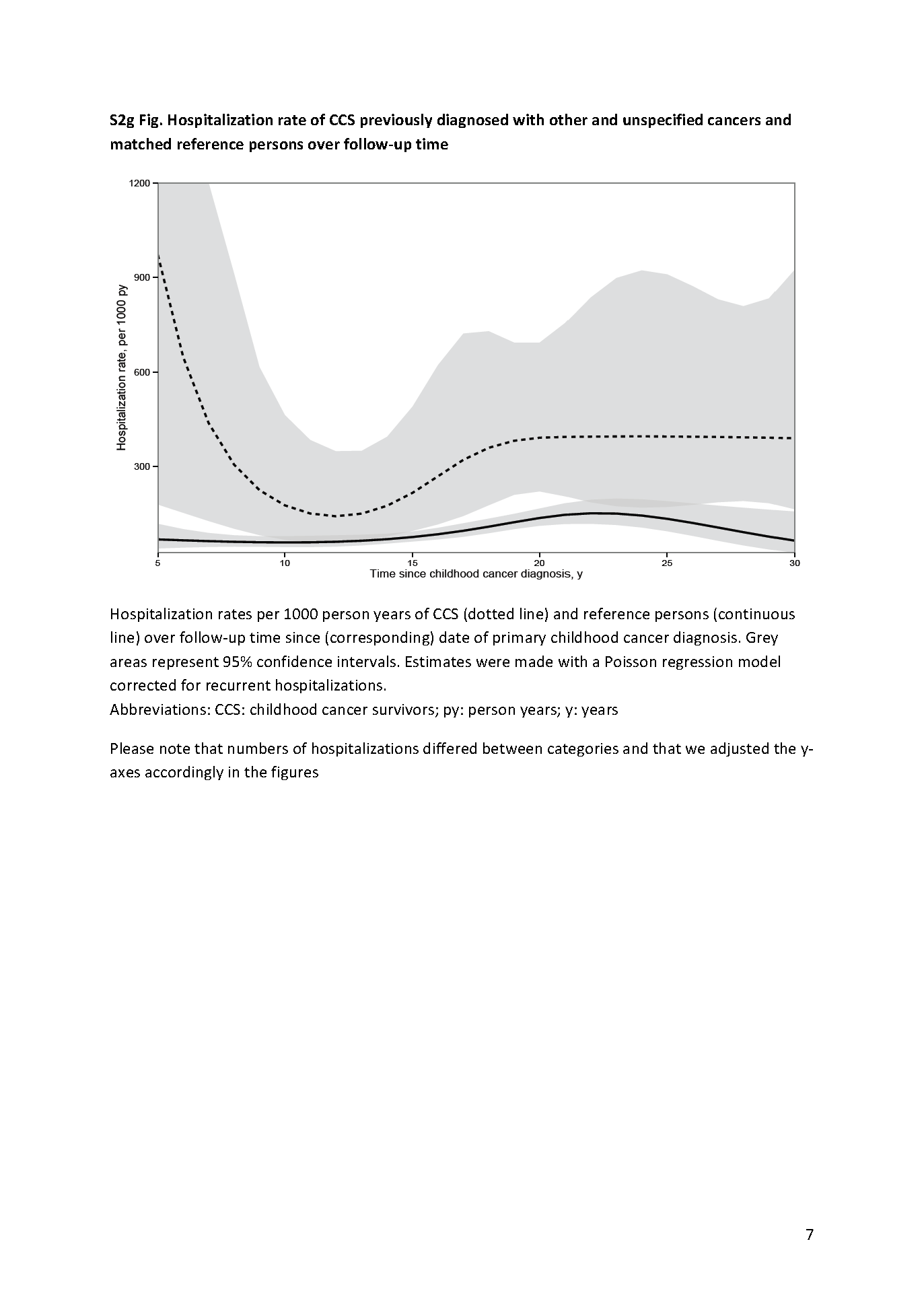

Supplement: S2 Fig — (A) Hospitalization rate of male CCS and reference persons over follow-up time. Hospitalization rates per 1000 person years of CCS (dotted line) and reference persons (continuous line) over follow-up time since (corresponding) date of primary childhood cancer diagnosis. Grey areas represent 95% confidence intervals. Estimates were made with a Poisson regression model corrected for recurrent hospitalizations. Abbreviations: CCS: childhood cancer survivors; py: person years; y: years. Please note that numbers of hospitalizations differed between categories and that we adjusted the y-axes accordingly in the figures. (B) Hospitalization rate of female CCS and reference persons over follow-up time. Hospitalization rates per 1000 person years of CCS (dotted line) and reference persons (continuous line) over follow-up time since (corresponding) date of primary childhood cancer diagnosis. Grey areas represent 95% confidence intervals. Estimates were made with a Poisson regression model corrected for recurrent hospitalizations. Abbreviations: CCS: childhood cancer survivors; py: person years; y: years. Please note that numbers of hospitalizations differed between categories and that we adjusted the y-axes accordingly in the figures. (C) Hospitalization rate of CCS previously diagnosed with leukemia or lymphoma and reference persons over follow-up time. Hospitalization rates per 1000 person years of CCS (dotted line) and reference persons (continuous line) over follow-up time since (corresponding) date of primary childhood cancer diagnosis. Grey areas represent 95% confidence intervals. Estimates were made with a Poisson regression model corrected for recurrent hospitalizations. Abbreviations: CCS: childhood cancer survivors; py: person years; y: years. Please note that numbers of hospitalizations differed between categories and that we adjusted the y-axes accordingly in the figures. (D) Hospitalization rate of CCS previously diagnosed with a central nervous system tumor and [file pone.0159518.s002.zip › S2_Fig/S2g_Fig.tif]

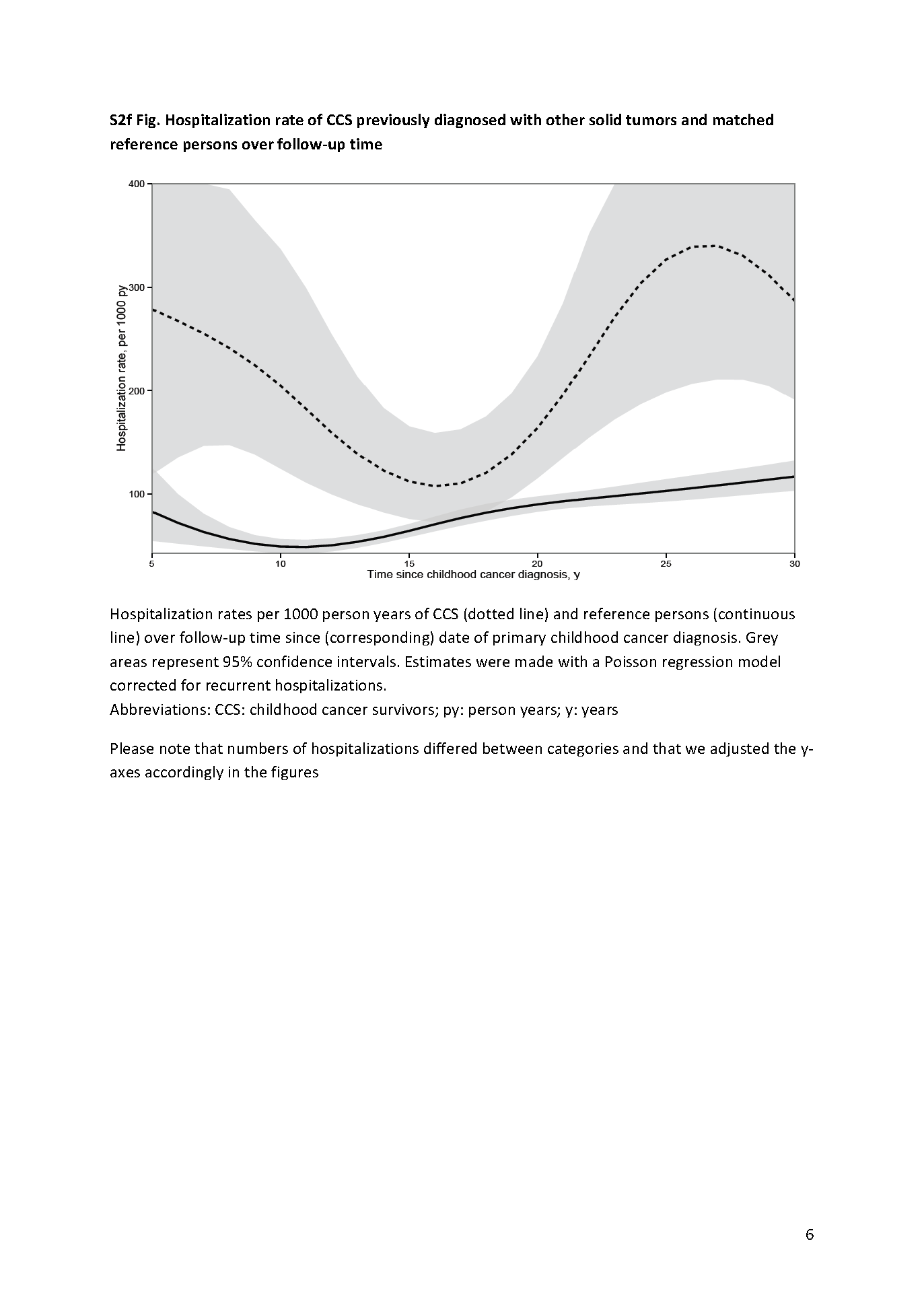

Supplement: S2 Fig — (A) Hospitalization rate of male CCS and reference persons over follow-up time. Hospitalization rates per 1000 person years of CCS (dotted line) and reference persons (continuous line) over follow-up time since (corresponding) date of primary childhood cancer diagnosis. Grey areas represent 95% confidence intervals. Estimates were made with a Poisson regression model corrected for recurrent hospitalizations. Abbreviations: CCS: childhood cancer survivors; py: person years; y: years. Please note that numbers of hospitalizations differed between categories and that we adjusted the y-axes accordingly in the figures. (B) Hospitalization rate of female CCS and reference persons over follow-up time. Hospitalization rates per 1000 person years of CCS (dotted line) and reference persons (continuous line) over follow-up time since (corresponding) date of primary childhood cancer diagnosis. Grey areas represent 95% confidence intervals. Estimates were made with a Poisson regression model corrected for recurrent hospitalizations. Abbreviations: CCS: childhood cancer survivors; py: person years; y: years. Please note that numbers of hospitalizations differed between categories and that we adjusted the y-axes accordingly in the figures. (C) Hospitalization rate of CCS previously diagnosed with leukemia or lymphoma and reference persons over follow-up time. Hospitalization rates per 1000 person years of CCS (dotted line) and reference persons (continuous line) over follow-up time since (corresponding) date of primary childhood cancer diagnosis. Grey areas represent 95% confidence intervals. Estimates were made with a Poisson regression model corrected for recurrent hospitalizations. Abbreviations: CCS: childhood cancer survivors; py: person years; y: years. Please note that numbers of hospitalizations differed between categories and that we adjusted the y-axes accordingly in the figures. (D) Hospitalization rate of CCS previously diagnosed with a central nervous system tumor and [file pone.0159518.s002.zip › S2_Fig/S2f_Fig.tif]

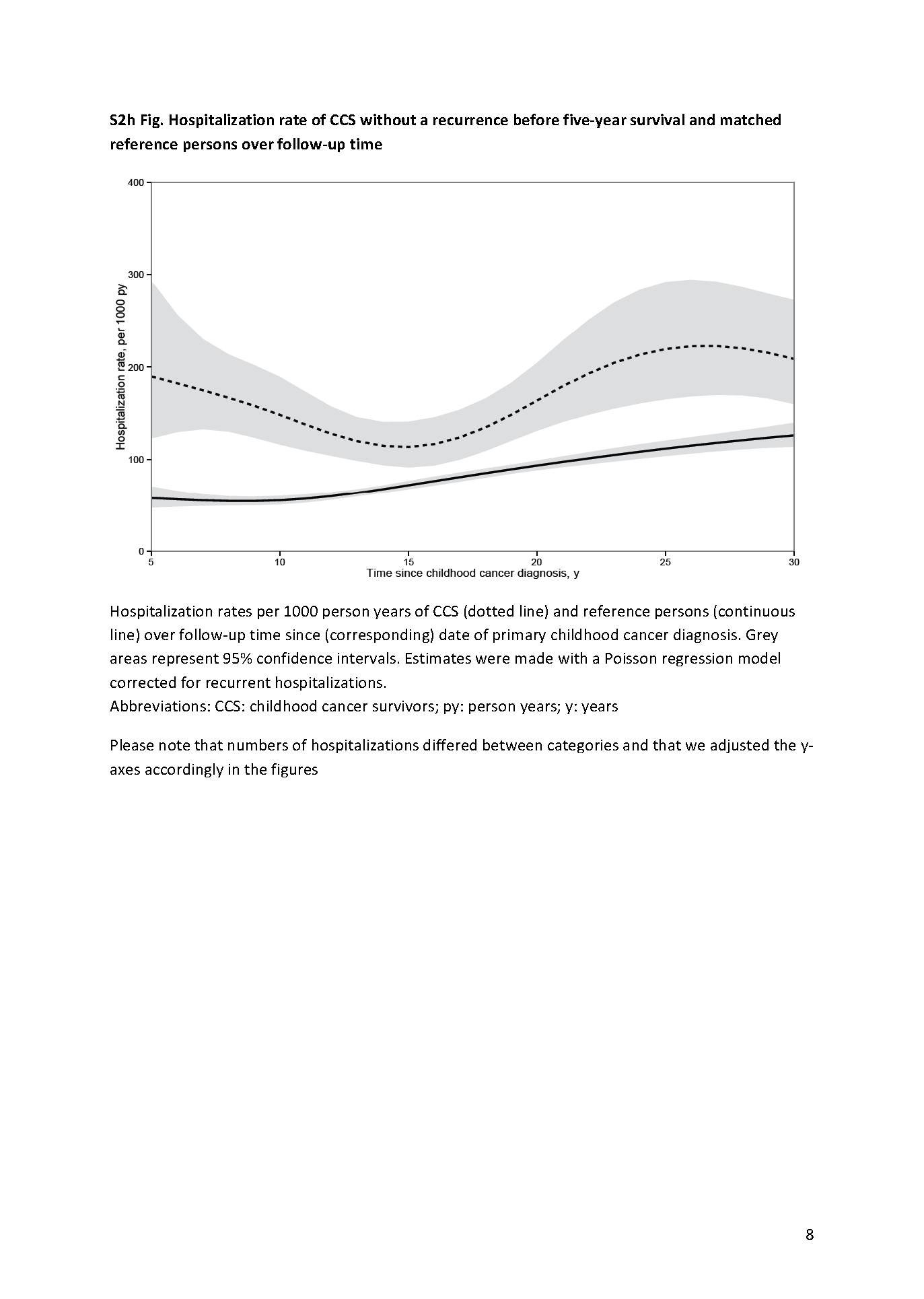

Supplement: S2 Fig — (A) Hospitalization rate of male CCS and reference persons over follow-up time. Hospitalization rates per 1000 person years of CCS (dotted line) and reference persons (continuous line) over follow-up time since (corresponding) date of primary childhood cancer diagnosis. Grey areas represent 95% confidence intervals. Estimates were made with a Poisson regression model corrected for recurrent hospitalizations. Abbreviations: CCS: childhood cancer survivors; py: person years; y: years. Please note that numbers of hospitalizations differed between categories and that we adjusted the y-axes accordingly in the figures. (B) Hospitalization rate of female CCS and reference persons over follow-up time. Hospitalization rates per 1000 person years of CCS (dotted line) and reference persons (continuous line) over follow-up time since (corresponding) date of primary childhood cancer diagnosis. Grey areas represent 95% confidence intervals. Estimates were made with a Poisson regression model corrected for recurrent hospitalizations. Abbreviations: CCS: childhood cancer survivors; py: person years; y: years. Please note that numbers of hospitalizations differed between categories and that we adjusted the y-axes accordingly in the figures. (C) Hospitalization rate of CCS previously diagnosed with leukemia or lymphoma and reference persons over follow-up time. Hospitalization rates per 1000 person years of CCS (dotted line) and reference persons (continuous line) over follow-up time since (corresponding) date of primary childhood cancer diagnosis. Grey areas represent 95% confidence intervals. Estimates were made with a Poisson regression model corrected for recurrent hospitalizations. Abbreviations: CCS: childhood cancer survivors; py: person years; y: years. Please note that numbers of hospitalizations differed between categories and that we adjusted the y-axes accordingly in the figures. (D) Hospitalization rate of CCS previously diagnosed with a central nervous system tumor and [file pone.0159518.s002.zip › S2_Fig/S2h_Fig.tif]

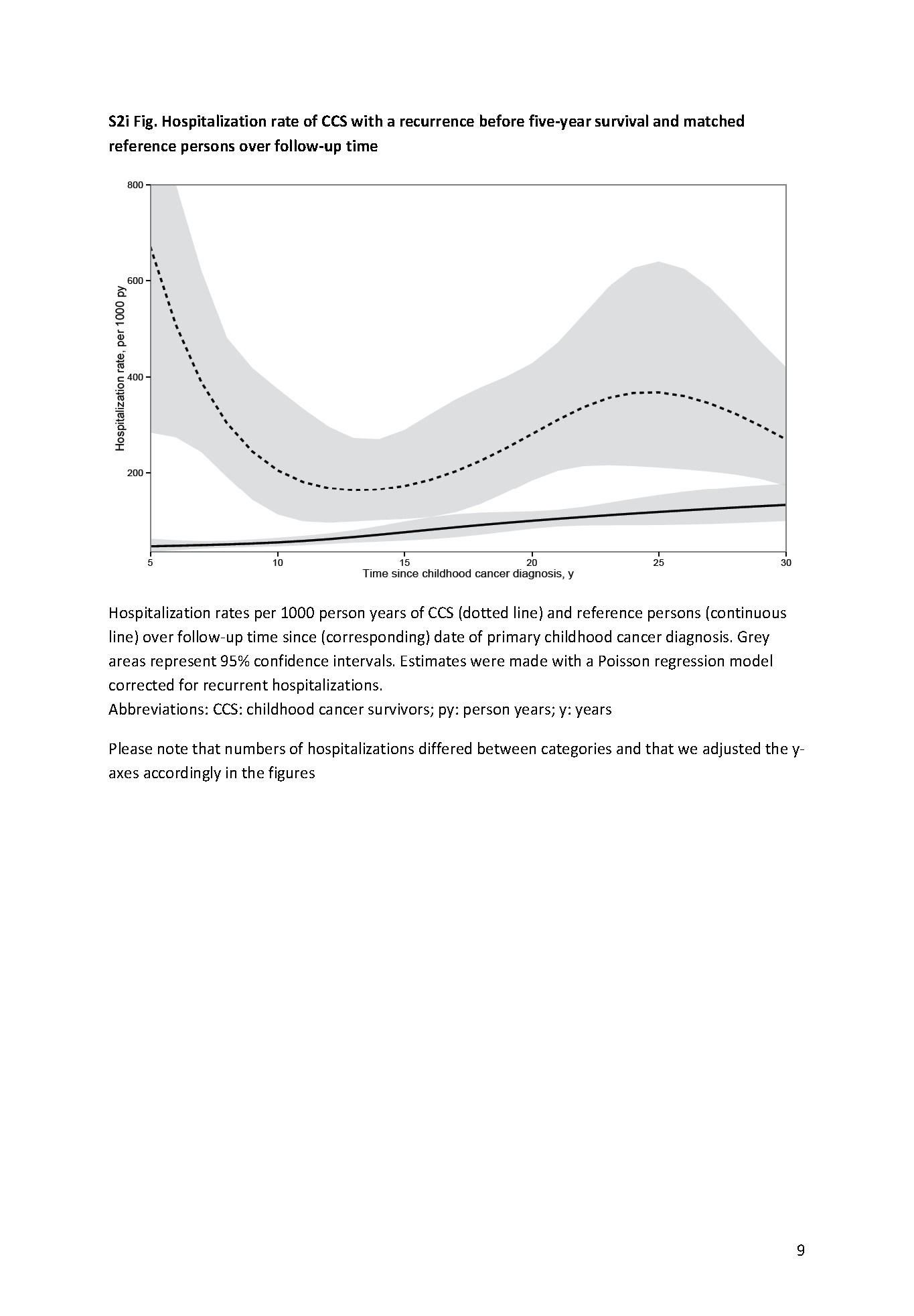

Supplement: S2 Fig — (A) Hospitalization rate of male CCS and reference persons over follow-up time. Hospitalization rates per 1000 person years of CCS (dotted line) and reference persons (continuous line) over follow-up time since (corresponding) date of primary childhood cancer diagnosis. Grey areas represent 95% confidence intervals. Estimates were made with a Poisson regression model corrected for recurrent hospitalizations. Abbreviations: CCS: childhood cancer survivors; py: person years; y: years. Please note that numbers of hospitalizations differed between categories and that we adjusted the y-axes accordingly in the figures. (B) Hospitalization rate of female CCS and reference persons over follow-up time. Hospitalization rates per 1000 person years of CCS (dotted line) and reference persons (continuous line) over follow-up time since (corresponding) date of primary childhood cancer diagnosis. Grey areas represent 95% confidence intervals. Estimates were made with a Poisson regression model corrected for recurrent hospitalizations. Abbreviations: CCS: childhood cancer survivors; py: person years; y: years. Please note that numbers of hospitalizations differed between categories and that we adjusted the y-axes accordingly in the figures. (C) Hospitalization rate of CCS previously diagnosed with leukemia or lymphoma and reference persons over follow-up time. Hospitalization rates per 1000 person years of CCS (dotted line) and reference persons (continuous line) over follow-up time since (corresponding) date of primary childhood cancer diagnosis. Grey areas represent 95% confidence intervals. Estimates were made with a Poisson regression model corrected for recurrent hospitalizations. Abbreviations: CCS: childhood cancer survivors; py: person years; y: years. Please note that numbers of hospitalizations differed between categories and that we adjusted the y-axes accordingly in the figures. (D) Hospitalization rate of CCS previously diagnosed with a central nervous system tumor and [file pone.0159518.s002.zip › S2_Fig/S2i_Fig.tif]

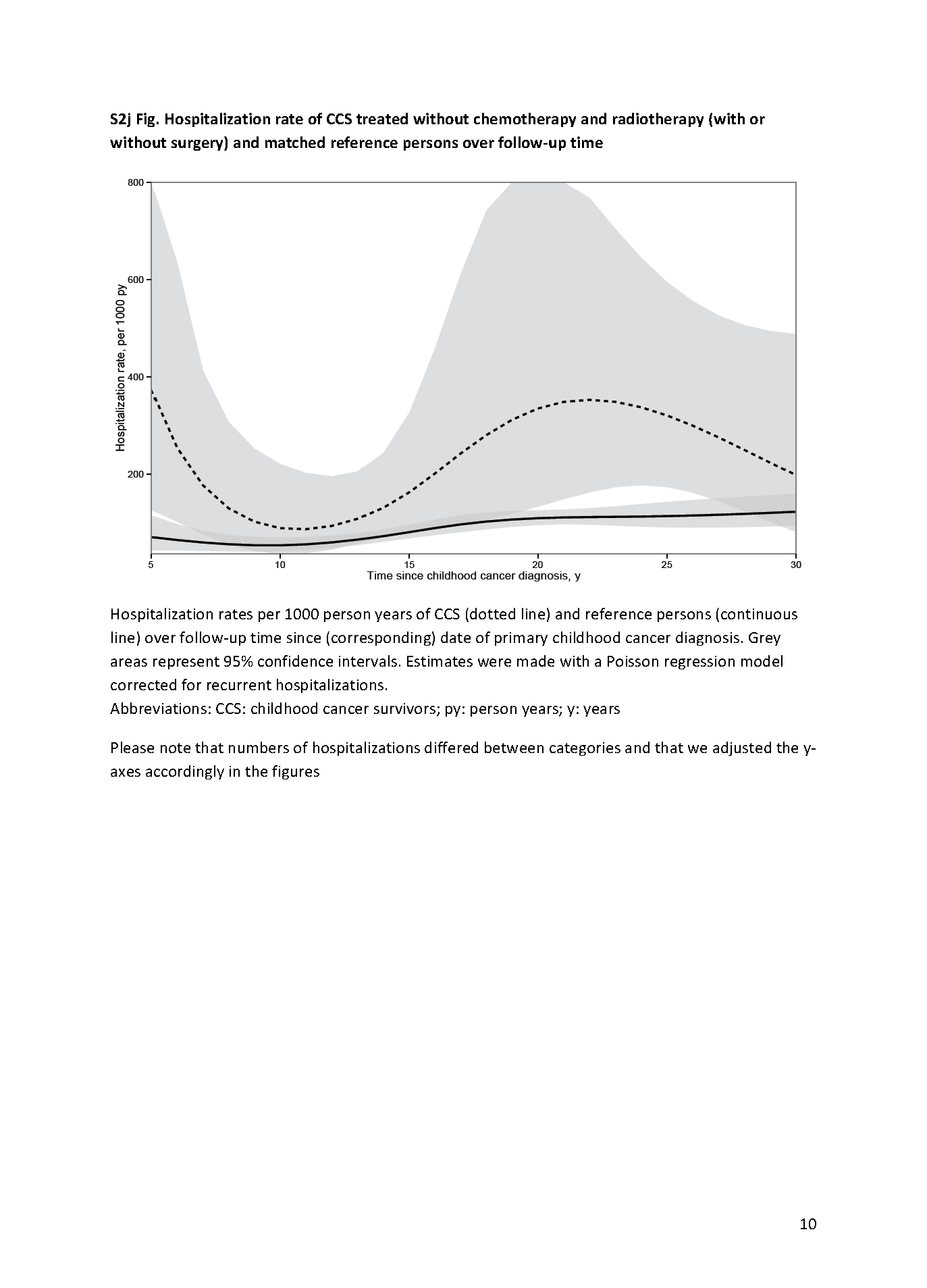

Supplement: S2 Fig — (A) Hospitalization rate of male CCS and reference persons over follow-up time. Hospitalization rates per 1000 person years of CCS (dotted line) and reference persons (continuous line) over follow-up time since (corresponding) date of primary childhood cancer diagnosis. Grey areas represent 95% confidence intervals. Estimates were made with a Poisson regression model corrected for recurrent hospitalizations. Abbreviations: CCS: childhood cancer survivors; py: person years; y: years. Please note that numbers of hospitalizations differed between categories and that we adjusted the y-axes accordingly in the figures. (B) Hospitalization rate of female CCS and reference persons over follow-up time. Hospitalization rates per 1000 person years of CCS (dotted line) and reference persons (continuous line) over follow-up time since (corresponding) date of primary childhood cancer diagnosis. Grey areas represent 95% confidence intervals. Estimates were made with a Poisson regression model corrected for recurrent hospitalizations. Abbreviations: CCS: childhood cancer survivors; py: person years; y: years. Please note that numbers of hospitalizations differed between categories and that we adjusted the y-axes accordingly in the figures. (C) Hospitalization rate of CCS previously diagnosed with leukemia or lymphoma and reference persons over follow-up time. Hospitalization rates per 1000 person years of CCS (dotted line) and reference persons (continuous line) over follow-up time since (corresponding) date of primary childhood cancer diagnosis. Grey areas represent 95% confidence intervals. Estimates were made with a Poisson regression model corrected for recurrent hospitalizations. Abbreviations: CCS: childhood cancer survivors; py: person years; y: years. Please note that numbers of hospitalizations differed between categories and that we adjusted the y-axes accordingly in the figures. (D) Hospitalization rate of CCS previously diagnosed with a central nervous system tumor and [file pone.0159518.s002.zip › S2_Fig/S2j_Fig.tif]

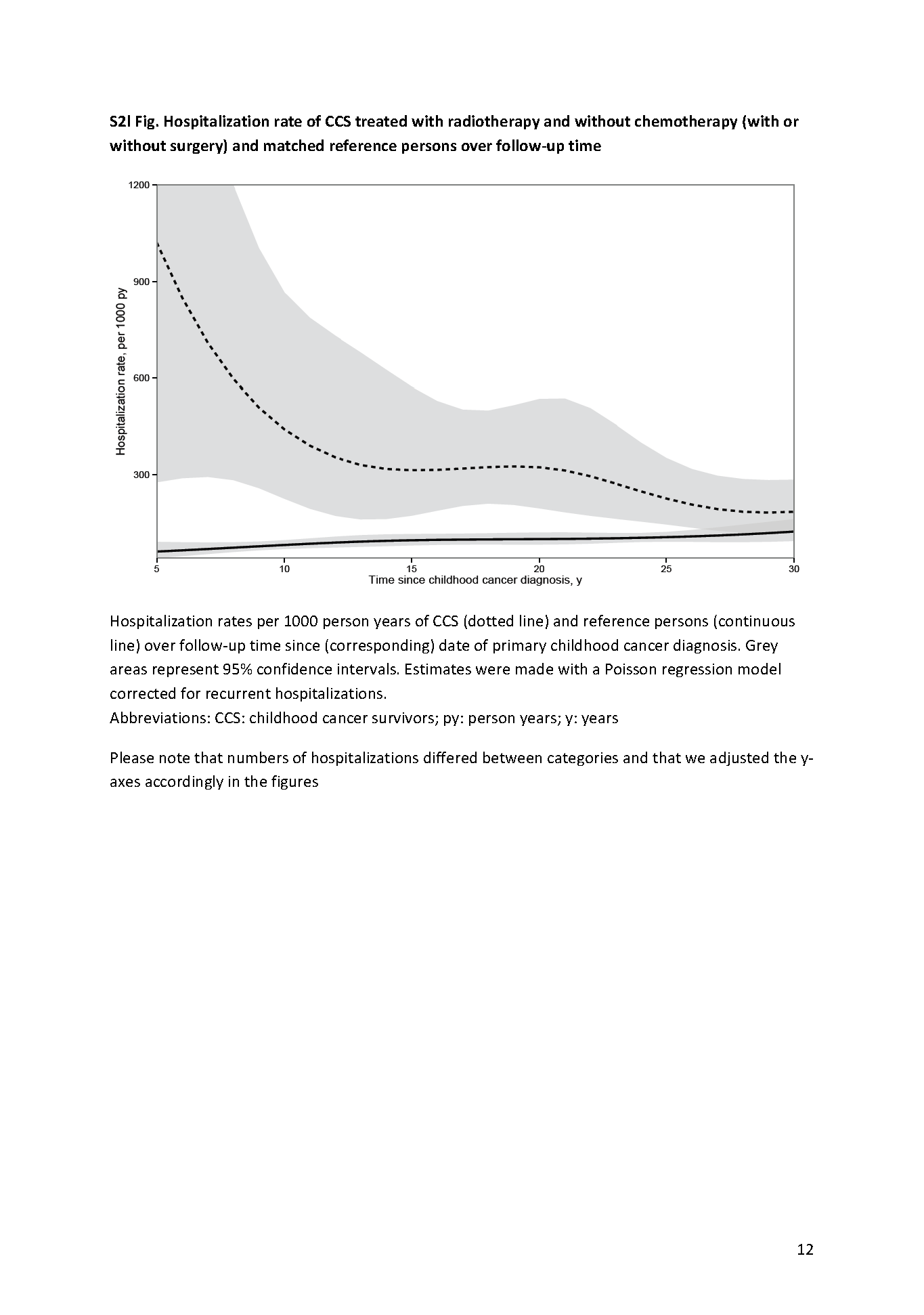

Supplement: S2 Fig — (A) Hospitalization rate of male CCS and reference persons over follow-up time. Hospitalization rates per 1000 person years of CCS (dotted line) and reference persons (continuous line) over follow-up time since (corresponding) date of primary childhood cancer diagnosis. Grey areas represent 95% confidence intervals. Estimates were made with a Poisson regression model corrected for recurrent hospitalizations. Abbreviations: CCS: childhood cancer survivors; py: person years; y: years. Please note that numbers of hospitalizations differed between categories and that we adjusted the y-axes accordingly in the figures. (B) Hospitalization rate of female CCS and reference persons over follow-up time. Hospitalization rates per 1000 person years of CCS (dotted line) and reference persons (continuous line) over follow-up time since (corresponding) date of primary childhood cancer diagnosis. Grey areas represent 95% confidence intervals. Estimates were made with a Poisson regression model corrected for recurrent hospitalizations. Abbreviations: CCS: childhood cancer survivors; py: person years; y: years. Please note that numbers of hospitalizations differed between categories and that we adjusted the y-axes accordingly in the figures. (C) Hospitalization rate of CCS previously diagnosed with leukemia or lymphoma and reference persons over follow-up time. Hospitalization rates per 1000 person years of CCS (dotted line) and reference persons (continuous line) over follow-up time since (corresponding) date of primary childhood cancer diagnosis. Grey areas represent 95% confidence intervals. Estimates were made with a Poisson regression model corrected for recurrent hospitalizations. Abbreviations: CCS: childhood cancer survivors; py: person years; y: years. Please note that numbers of hospitalizations differed between categories and that we adjusted the y-axes accordingly in the figures. (D) Hospitalization rate of CCS previously diagnosed with a central nervous system tumor and [file pone.0159518.s002.zip › S2_Fig/S2l_Fig.tif]

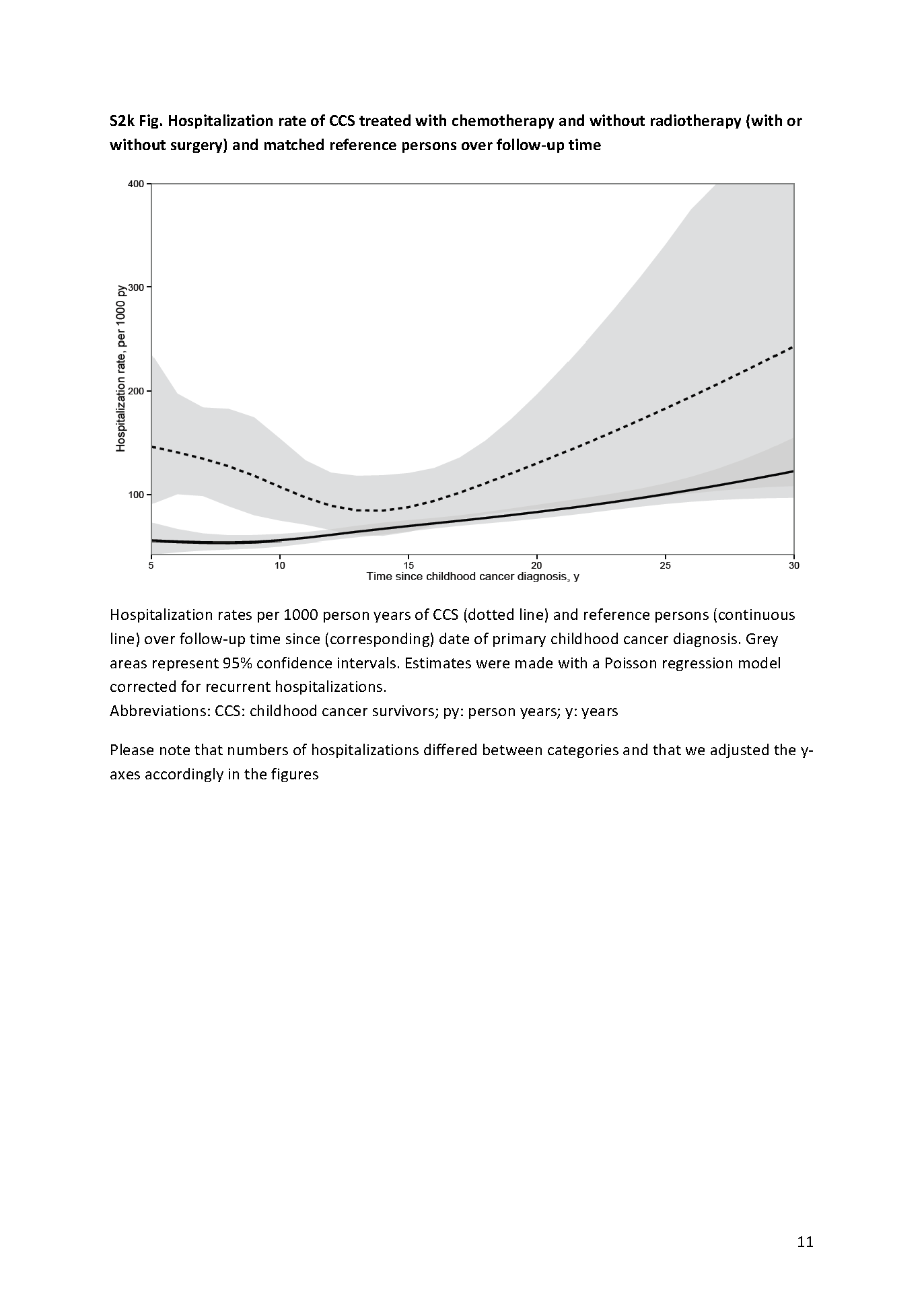

Supplement: S2 Fig — (A) Hospitalization rate of male CCS and reference persons over follow-up time. Hospitalization rates per 1000 person years of CCS (dotted line) and reference persons (continuous line) over follow-up time since (corresponding) date of primary childhood cancer diagnosis. Grey areas represent 95% confidence intervals. Estimates were made with a Poisson regression model corrected for recurrent hospitalizations. Abbreviations: CCS: childhood cancer survivors; py: person years; y: years. Please note that numbers of hospitalizations differed between categories and that we adjusted the y-axes accordingly in the figures. (B) Hospitalization rate of female CCS and reference persons over follow-up time. Hospitalization rates per 1000 person years of CCS (dotted line) and reference persons (continuous line) over follow-up time since (corresponding) date of primary childhood cancer diagnosis. Grey areas represent 95% confidence intervals. Estimates were made with a Poisson regression model corrected for recurrent hospitalizations. Abbreviations: CCS: childhood cancer survivors; py: person years; y: years. Please note that numbers of hospitalizations differed between categories and that we adjusted the y-axes accordingly in the figures. (C) Hospitalization rate of CCS previously diagnosed with leukemia or lymphoma and reference persons over follow-up time. Hospitalization rates per 1000 person years of CCS (dotted line) and reference persons (continuous line) over follow-up time since (corresponding) date of primary childhood cancer diagnosis. Grey areas represent 95% confidence intervals. Estimates were made with a Poisson regression model corrected for recurrent hospitalizations. Abbreviations: CCS: childhood cancer survivors; py: person years; y: years. Please note that numbers of hospitalizations differed between categories and that we adjusted the y-axes accordingly in the figures. (D) Hospitalization rate of CCS previously diagnosed with a central nervous system tumor and [file pone.0159518.s002.zip › S2_Fig/S2k_Fig.tif]

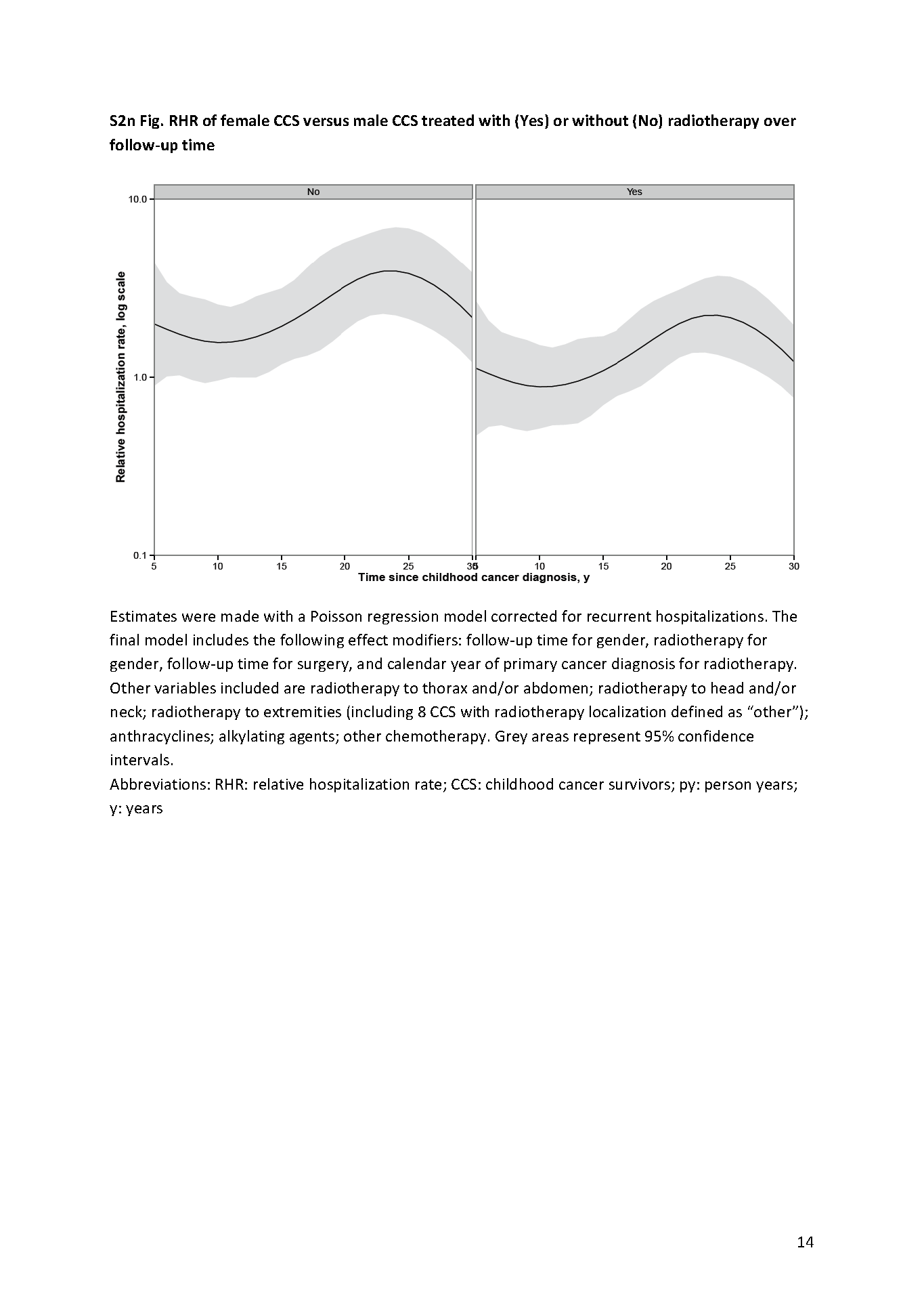

Supplement: S2 Fig — (A) Hospitalization rate of male CCS and reference persons over follow-up time. Hospitalization rates per 1000 person years of CCS (dotted line) and reference persons (continuous line) over follow-up time since (corresponding) date of primary childhood cancer diagnosis. Grey areas represent 95% confidence intervals. Estimates were made with a Poisson regression model corrected for recurrent hospitalizations. Abbreviations: CCS: childhood cancer survivors; py: person years; y: years. Please note that numbers of hospitalizations differed between categories and that we adjusted the y-axes accordingly in the figures. (B) Hospitalization rate of female CCS and reference persons over follow-up time. Hospitalization rates per 1000 person years of CCS (dotted line) and reference persons (continuous line) over follow-up time since (corresponding) date of primary childhood cancer diagnosis. Grey areas represent 95% confidence intervals. Estimates were made with a Poisson regression model corrected for recurrent hospitalizations. Abbreviations: CCS: childhood cancer survivors; py: person years; y: years. Please note that numbers of hospitalizations differed between categories and that we adjusted the y-axes accordingly in the figures. (C) Hospitalization rate of CCS previously diagnosed with leukemia or lymphoma and reference persons over follow-up time. Hospitalization rates per 1000 person years of CCS (dotted line) and reference persons (continuous line) over follow-up time since (corresponding) date of primary childhood cancer diagnosis. Grey areas represent 95% confidence intervals. Estimates were made with a Poisson regression model corrected for recurrent hospitalizations. Abbreviations: CCS: childhood cancer survivors; py: person years; y: years. Please note that numbers of hospitalizations differed between categories and that we adjusted the y-axes accordingly in the figures. (D) Hospitalization rate of CCS previously diagnosed with a central nervous system tumor and [file pone.0159518.s002.zip › S2_Fig/S2n_Fig.tif]

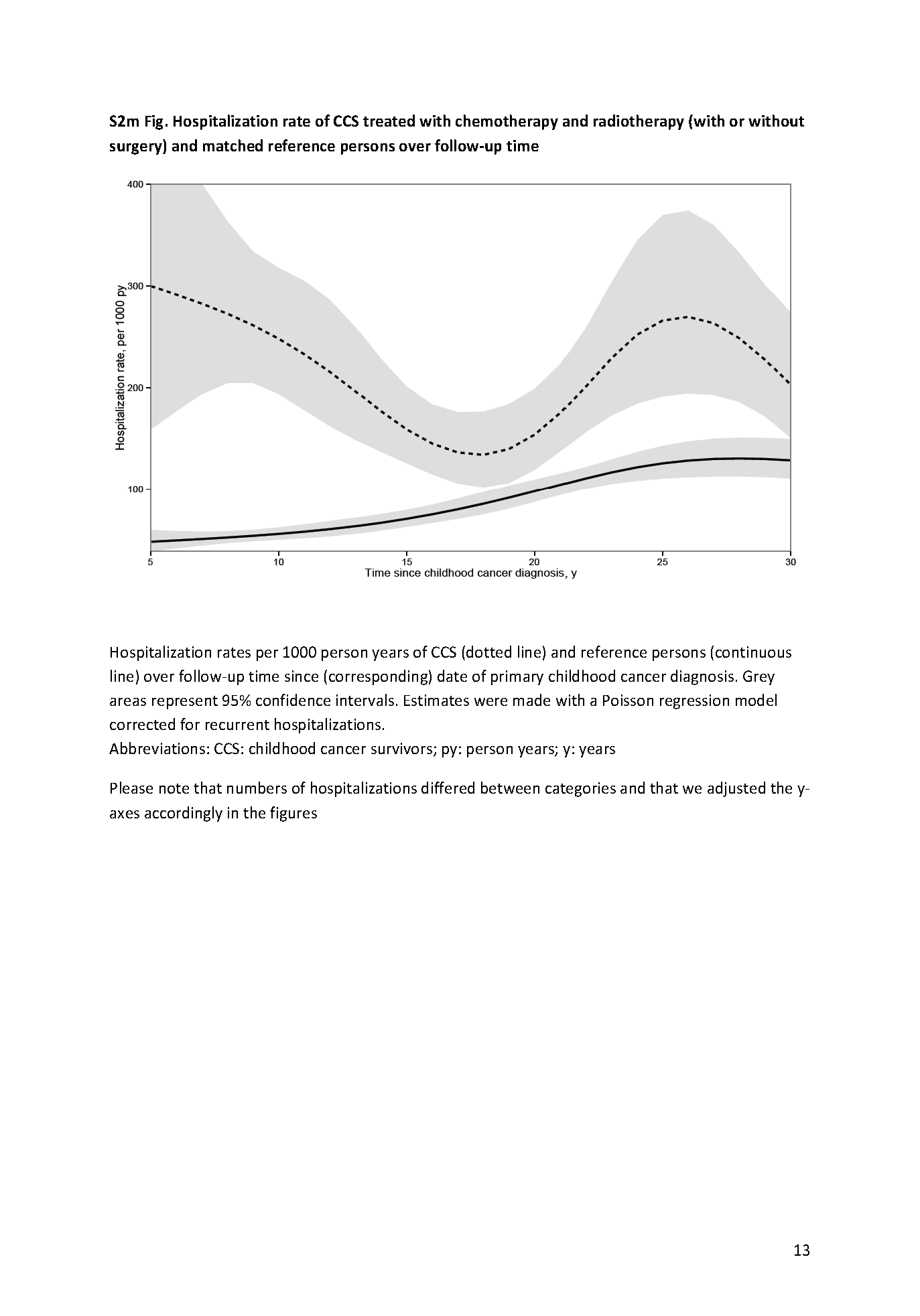

Supplement: S2 Fig — (A) Hospitalization rate of male CCS and reference persons over follow-up time. Hospitalization rates per 1000 person years of CCS (dotted line) and reference persons (continuous line) over follow-up time since (corresponding) date of primary childhood cancer diagnosis. Grey areas represent 95% confidence intervals. Estimates were made with a Poisson regression model corrected for recurrent hospitalizations. Abbreviations: CCS: childhood cancer survivors; py: person years; y: years. Please note that numbers of hospitalizations differed between categories and that we adjusted the y-axes accordingly in the figures. (B) Hospitalization rate of female CCS and reference persons over follow-up time. Hospitalization rates per 1000 person years of CCS (dotted line) and reference persons (continuous line) over follow-up time since (corresponding) date of primary childhood cancer diagnosis. Grey areas represent 95% confidence intervals. Estimates were made with a Poisson regression model corrected for recurrent hospitalizations. Abbreviations: CCS: childhood cancer survivors; py: person years; y: years. Please note that numbers of hospitalizations differed between categories and that we adjusted the y-axes accordingly in the figures. (C) Hospitalization rate of CCS previously diagnosed with leukemia or lymphoma and reference persons over follow-up time. Hospitalization rates per 1000 person years of CCS (dotted line) and reference persons (continuous line) over follow-up time since (corresponding) date of primary childhood cancer diagnosis. Grey areas represent 95% confidence intervals. Estimates were made with a Poisson regression model corrected for recurrent hospitalizations. Abbreviations: CCS: childhood cancer survivors; py: person years; y: years. Please note that numbers of hospitalizations differed between categories and that we adjusted the y-axes accordingly in the figures. (D) Hospitalization rate of CCS previously diagnosed with a central nervous system tumor and [file pone.0159518.s002.zip › S2_Fig/S2m_Fig.tif]

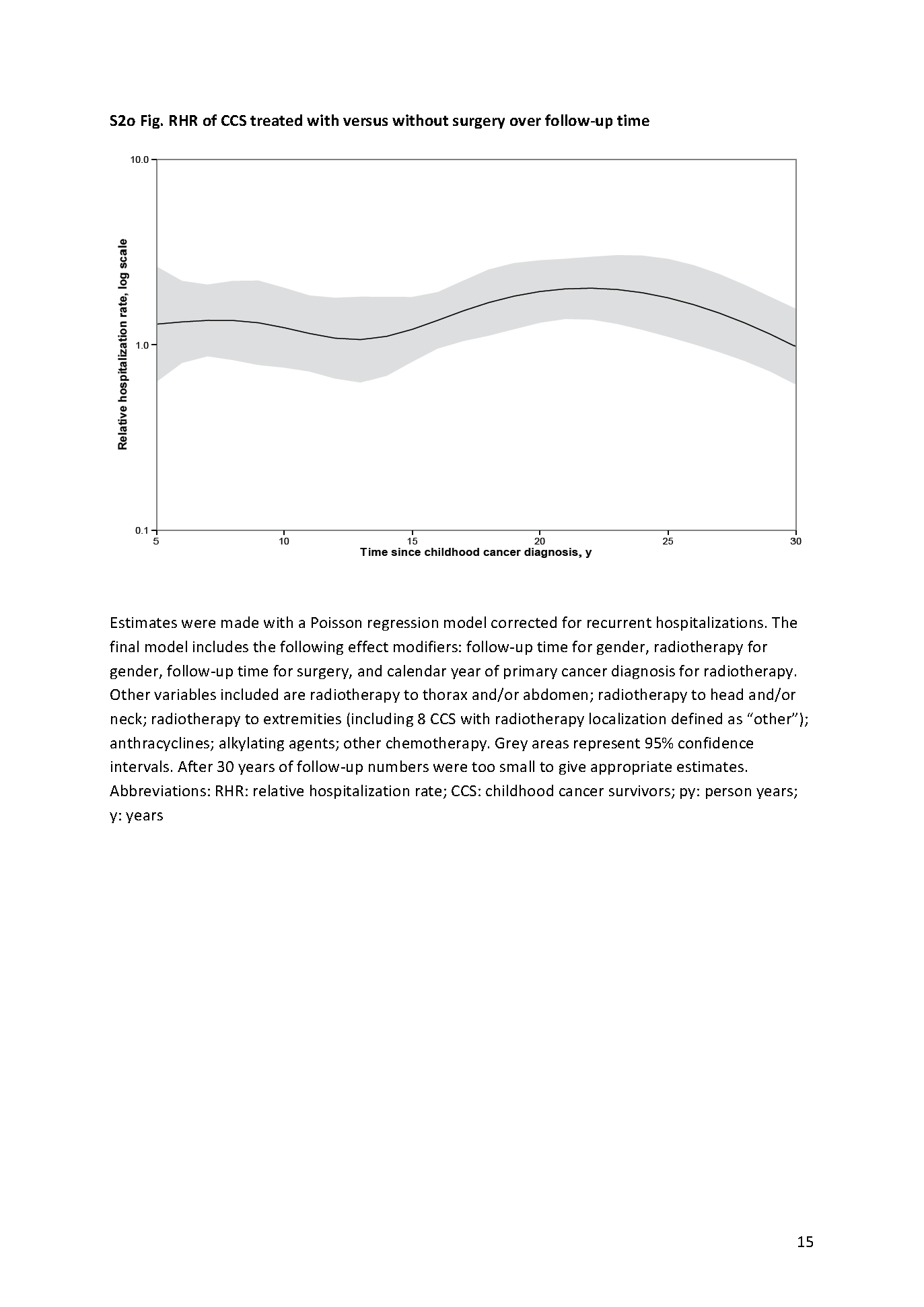

Supplement: S2 Fig — (A) Hospitalization rate of male CCS and reference persons over follow-up time. Hospitalization rates per 1000 person years of CCS (dotted line) and reference persons (continuous line) over follow-up time since (corresponding) date of primary childhood cancer diagnosis. Grey areas represent 95% confidence intervals. Estimates were made with a Poisson regression model corrected for recurrent hospitalizations. Abbreviations: CCS: childhood cancer survivors; py: person years; y: years. Please note that numbers of hospitalizations differed between categories and that we adjusted the y-axes accordingly in the figures. (B) Hospitalization rate of female CCS and reference persons over follow-up time. Hospitalization rates per 1000 person years of CCS (dotted line) and reference persons (continuous line) over follow-up time since (corresponding) date of primary childhood cancer diagnosis. Grey areas represent 95% confidence intervals. Estimates were made with a Poisson regression model corrected for recurrent hospitalizations. Abbreviations: CCS: childhood cancer survivors; py: person years; y: years. Please note that numbers of hospitalizations differed between categories and that we adjusted the y-axes accordingly in the figures. (C) Hospitalization rate of CCS previously diagnosed with leukemia or lymphoma and reference persons over follow-up time. Hospitalization rates per 1000 person years of CCS (dotted line) and reference persons (continuous line) over follow-up time since (corresponding) date of primary childhood cancer diagnosis. Grey areas represent 95% confidence intervals. Estimates were made with a Poisson regression model corrected for recurrent hospitalizations. Abbreviations: CCS: childhood cancer survivors; py: person years; y: years. Please note that numbers of hospitalizations differed between categories and that we adjusted the y-axes accordingly in the figures. (D) Hospitalization rate of CCS previously diagnosed with a central nervous system tumor and [file pone.0159518.s002.zip › S2_Fig/S2o_Fig.tif]

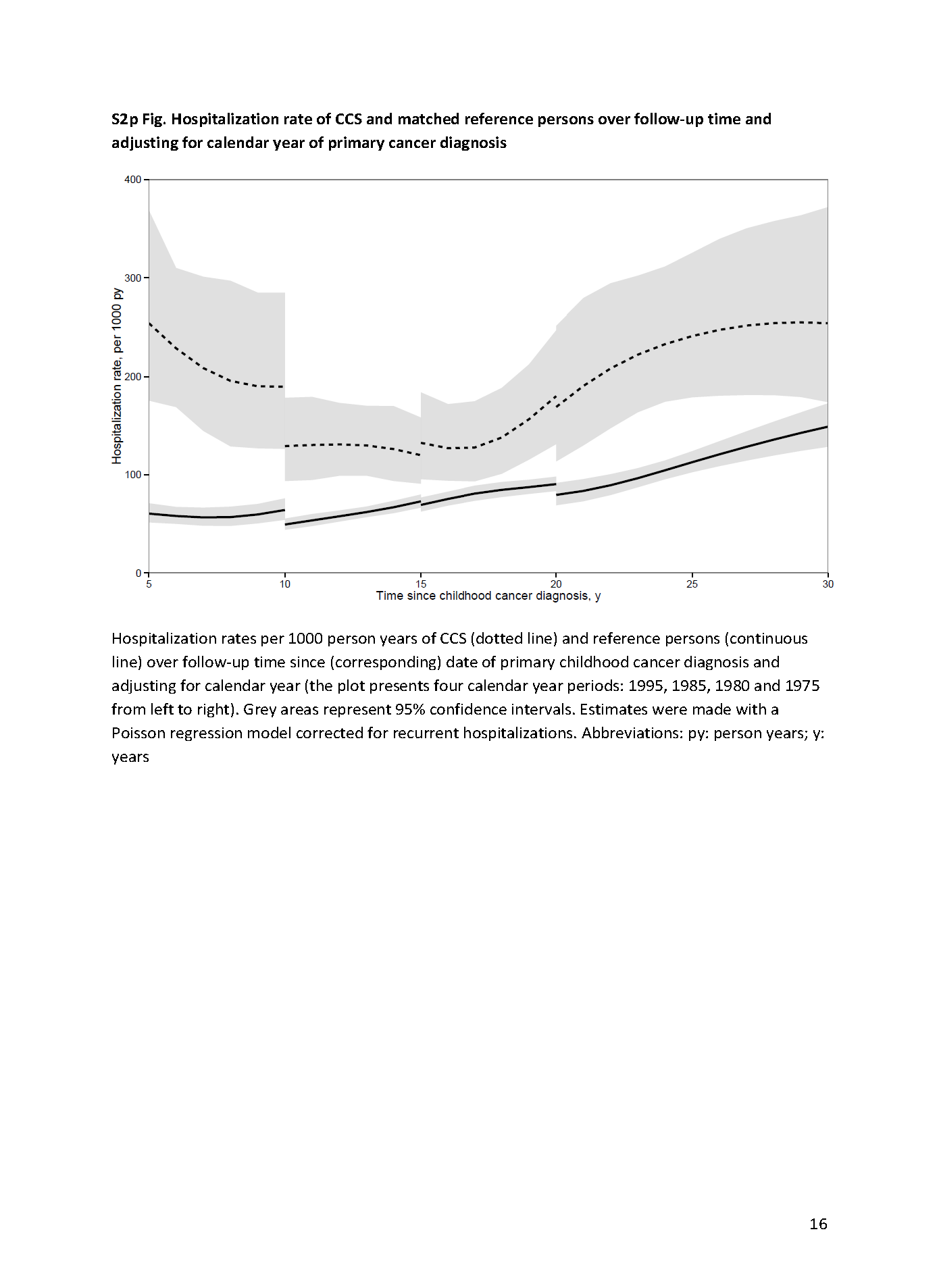

Supplement: S2 Fig — (A) Hospitalization rate of male CCS and reference persons over follow-up time. Hospitalization rates per 1000 person years of CCS (dotted line) and reference persons (continuous line) over follow-up time since (corresponding) date of primary childhood cancer diagnosis. Grey areas represent 95% confidence intervals. Estimates were made with a Poisson regression model corrected for recurrent hospitalizations. Abbreviations: CCS: childhood cancer survivors; py: person years; y: years. Please note that numbers of hospitalizations differed between categories and that we adjusted the y-axes accordingly in the figures. (B) Hospitalization rate of female CCS and reference persons over follow-up time. Hospitalization rates per 1000 person years of CCS (dotted line) and reference persons (continuous line) over follow-up time since (corresponding) date of primary childhood cancer diagnosis. Grey areas represent 95% confidence intervals. Estimates were made with a Poisson regression model corrected for recurrent hospitalizations. Abbreviations: CCS: childhood cancer survivors; py: person years; y: years. Please note that numbers of hospitalizations differed between categories and that we adjusted the y-axes accordingly in the figures. (C) Hospitalization rate of CCS previously diagnosed with leukemia or lymphoma and reference persons over follow-up time. Hospitalization rates per 1000 person years of CCS (dotted line) and reference persons (continuous line) over follow-up time since (corresponding) date of primary childhood cancer diagnosis. Grey areas represent 95% confidence intervals. Estimates were made with a Poisson regression model corrected for recurrent hospitalizations. Abbreviations: CCS: childhood cancer survivors; py: person years; y: years. Please note that numbers of hospitalizations differed between categories and that we adjusted the y-axes accordingly in the figures. (D) Hospitalization rate of CCS previously diagnosed with a central nervous system tumor and [file pone.0159518.s002.zip › S2_Fig/S2p_Fig.tif]
