# Supplementary material for: High Hospitalization Rates in Survivors of Childhood Cancer: A Longitudinal Follow-Up Study Using Medical Record Linkage
Source: PLoS One. 2016 Jul 19;11(7):e0159518. doi: 10.1371/journal.pone.0159518 (PMC4951023; doi:10.1371/journal.pone.0159518)
Supplement: S1 Methods — (DOCX) [file pone.0159518.s003.docx]

**S1 Methods. Details on censoring of CCS and corresponding reference persons**.

After censoring CCS (and corresponding reference persons) at late primary cancer recurrences or primary cancer recurrence therapy, 1292 CCS contributed to unique follow-up time. Ninety CCS did not contribute to any follow-up time in our analyses: 39 CCS who were on on-going cancer therapy for a primary cancer recurrence and 51 of the 86 CCS who developed a primary cancer recurrence before 1995. The remaining 86-51=35 were censored at first cancer recurrence.
