# Supplementary material for: High Hospitalization Rates in Survivors of Childhood Cancer: A Longitudinal Follow-Up Study Using Medical Record Linkage
Source: PLoS One. 2016 Jul 19;11(7):e0159518. doi: 10.1371/journal.pone.0159518 (PMC4951023; doi:10.1371/journal.pone.0159518)
Supplement: S1 Table — (DOCX) [file pone.0159518.s004.docx]

**S1 Table. Calendar year of primary cancer diagnosis and corresponding follow-up time since childhood cancer diagnosis and attained age at the end of follow-up.**

| **Calendar year of primary cancer diagnosis** | **Follow-up time since childhood cancer diagnosis (years)** | **Attained age at the end of follow-up (years)** |
| --- | --- | --- |
| 1995 | 5-11 | 5-29 |
| 1985 | 10-21 | 10-39 |
| 1980 | 15-26 | 15-44 |
| 1975 | 20-31 | 20-49 |
